# Use of Ionic Liquids and Co-Solvents for Synthesis of Thin-Film Composite Membranes

**DOI:** 10.3390/membranes11040297

**Published:** 2021-04-20

**Authors:** Peter-Renaat Van den Mooter, Liridona Dedvukaj, Ivo F. J. Vankelecom

**Affiliations:** Membrane Technology Group (MTG)—Division cMACS—Faculty of Bioscience Engineering KU Leuven, Celestijnenlaan 200F, Box 2454, 3001 Leuven, Belgium; peter.vandenmooter1@kuleuven.be

**Keywords:** ionic liquids, TFC membranes, interfacial polymerization, polyamide, epoxide, STNF

## Abstract

Polyamide (PA) thin-film composite (TFC) membranes are commonly applied in reversed osmosis (RO) and nanofiltration (NF) applications due to their thin, dense top-layer, and high selectivity. Recently, the conventional organic phase (i.e., hexane) during interfacial polymerization (IP) was replaced by less toxic ionic liquids (ILs) which led to excellent membrane performances. As the high price of most ILs limits their up-scaling, the potential use of inexpensive Aliquat was investigated in this study. The thin-film composite (TFC) membranes were optimized to remove flavor compounds, i.e., ethyl acetate (EA) and isoamyl acetate (IA), from a fermentation broth. A multi-parameter optimization was set-up involving type of support, reaction time for IP, water content of Aliquat, and concentration of both monomers m-phenylenediamine (MPD) and trimesoylchloride (TMC). The membranes prepared using Aliquat showed similar fluxes as those prepared from a reference IL 1-butyl-1-methylpyrrolidinium bis(trifluoromethylsulfonyl)imide ([C_4_mpyr][Tf_2_N]) but with better EA and IA retentions, even better than for a commercial RO membrane (GEA type AF). Finally, the recently introduced epoxide-curing of Bisphenol A diglycidyl ether (BADGE) with 1,6-hexanediamine (HDA) was investigated using Aliquat as organic phase. It is the first time this type of IP was performed in combination with an IL as organic phase. The resulting membrane was used in the filtration of a 35 µM Rose Bengal (RB) in 20 wt% dimethylformamide/ water (DMF/H_2_O) feed mixture. A well-crosslinked poly(*β*-alkanolamine) film was obtained with a > 97% retention.

## 1. Introduction

**Figure 1:**
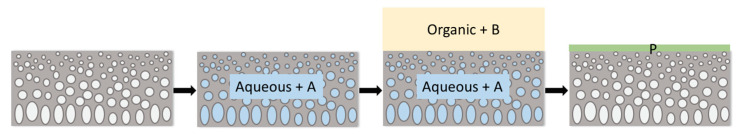
Schematic representation of the interfacial polymerization technique. A and B are monomers and P is the polymer formed at the interface. (adapted from [1]).

**Figure 2:**
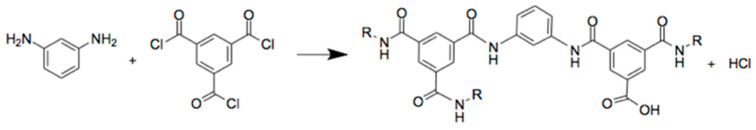
Polycondensation reaction between m-phenylenediamine (MPD) and trimesoylchloride (TMC) with the formation of polyamide (PA).

Thin film composite (TFC) membranes consist of an ultrathin functional layer on top of a porous support [1,2,3,4]. The porous supports, typically consisting of polysulfone (PSf) for aqueous applications, are made via non-solvent induced phase separation (NIPS) [5]. The thin films are usually obtained via interfacial polymerization (IP) [6,7]. Here, the support, impregnated by an aqueous phase, is brought in contact with an immiscible organic phase, both containing a different monomer (Figure 1). Next, polymerization of the top-layer occurs at the interface. Common monomers used are *meta*-phenylene diamine (MPD) for the aqueous phase and trimesoyl chloride (TMC) for the organic phase, generating a very thin, dense, and highly cross-linked polyamide (PA) top-layer (Figure 2). These membranes are mostly applied in pressure-driven membrane processes, such as reverse osmosis (RO), nanofiltration (NF), or solvent-tolerant or solvent-resistant nanofiltration (STNF and SRNF, respectively), to retain low molecular weight components [4,5,6,7,8,9]. Commercial PA-TFC membranes show very high salt rejections of 99.4–99.8% [4].

Since the conventional system for synthesizing TFC membranes uses toxic volatile organics, such as hexane, research is done to replace them by less volatile and less toxic solvents. Over the last two decades, ionic liquids (ILs) have gained interest because of their special properties and characteristics [10,11]. ILs are salts that are termed as green solvents because of their low vapor pressure. However, ILs as a class do not completely meet the 12 Principles of Green Chemistry since some ILs have a certain level of toxicity which increases with increasing alkyl chain length [11,12,13].

ILs are interesting because of their chemical and thermal stability and tuneable solvent properties [10,11,14,15]. Within membrane synthesis, ILs have successfully been introduced as casting solvent to prepare membranes via NIPS [14,15,16,17]. More recently, ILs were used to substitute hexane as organic phase for IP [18,19]. The IL properties hold a major influence on the properties of the selective layer, as the top-layer formation is assumed to take place in the organic phase. The optimal concentration of the more toxic monomer during IP decreased 20-fold. Additionally, a thinner and smoother selective top-layer was obtained, resulting in higher membrane fluxes and less fouling. Moreover, ILs exhibited surfactant properties which eliminated the need for surfactants and catalysts [18,19]. Nevertheless, ILs can be rather expensive which might limit their up-scaling, even though the possibility to recycle them was proven [10,15,16,17,20,21].

Recently, epoxide chemistry has been introduced to achieve exceptionally stable (SR)NF membranes. Epoxy-based membranes were obtained by using the concept of conventional IP. First, a cross-linked polyimide (XL-PI) support was impregnated with an aqueous amine solution (e.g., *N*,*N*,*N*′,*N*′-tetramethyl-1,6-hexanediamine (TMHD)) and subsequently contacted with an epoxide solution in toluene. A highly cross-linked polymer was formed displaying increased mechanical and chemical resistance [22,23,24].

The aim of this work was to study the feasibility of replacing the conventionally used and harmful organic solvents by an economically interesting IL (i.e., Aliquat) and to optimize the synthesis of scalable TFC PA-membranes with this IL. Additionally, the selected IL was further combined with epoxy chemistry to produce TFC membranes. Different synthesis parameters were screened, i.e., support type, reaction time for IP, and concentration of the monomers. The PA-TFC membranes were tested in a high-alcoholic synthetic feed mixture to remove ethylacetate (EA) and isoamyl acetate (IA) as flavor compounds, as typically found in beverage and food industry. Above a threshold concentration, these esters are known to create an unpleasant fruity and banana smell and taste in beverages [25,26,27,28,29,30]. The epoxy-based TFC membranes were tested for their (ST)NF performance removing a Rose Bengal (RB) (Figure S21) from a DMF/H_2_O feed.

## 2. Materials and Methods

### 2.1. Materials

**Figure 3:**
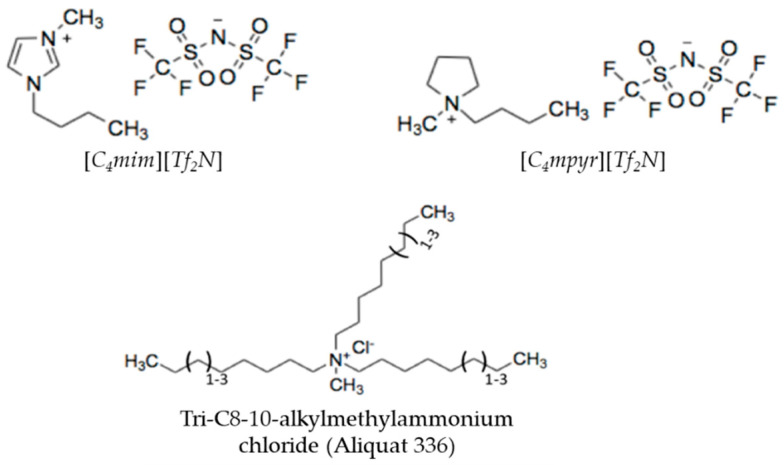
Chemical structures of [*C*_4_*mim*][*Tf_2_N*], [*C*_4_*mpyr*][*Tf_2_N*], and Aliquat.

Commercially available polysulfone (PSf, Udel^®^ P-1700, Solvay, Beveren, Belgium) and polyimide (PI, Matrimid^®^ 5218, Huntsman Corporation, Everberg, Belgium) were kindly provided by Solvay and Huntsman, respectively. The pellets were dried overnight at 100 °C prior to use. Non-woven polyproplylene/polyethylene (PP/PE) (Novatexx 2471) was purchased from Freudenberg Filtration (Weinheim, GE). Tetrahydrofuran (THF, > 99.9%, Sigma-Aldrich, Overijse, Belgium), dimethylformamide (DMF, 99%, Acros Organics, Leuven, BE), 1-methyl-2-pyrrolidone (NMP, 99%, Acros Organics, Leuven, Belgium), Hexanediamine (HDA, 99.99%, Fisher Scientific, Loughborough, UK), M-phenylenediamine (MPD, > 99%, Acros Organics, Leuven, Belgium), Trimesoyl chloride (TMC, 98%, Acros Organics), n-hexane (> 97%, Honeywell, Loughborough, UK), Acetonitrile (ACN, 99.99%, Fisher Scientific, Loughborough, UK), Acetone (Technical, VWR, Leuven, Belgium) were used for membrane synthesis. Epoxy resin Bisphenol A diglycidyl ether (BADGE, > 99%, Sigma Aldrich, Overijse, Belgium) was used as received without further purification (Figure S1a). ILs 1-Butyl-3-methylimidazolium bis(trifluoromethylsulfonyl)imide ([*C*_4_*mim*][*Tf_2_N*], 99+%, Iolitec, Heilbronn, GE), 1-butyl-1-methylpyrrolidinium bis(trifluoromethylsulfonyl)imide ([*C*_4_*mpyr*][*Tf_2_N*], 99+%, Iolitec, Heilbronn, GE) and trioctylmethylammonium chloride (Aliquat^®^ 336 TG (here referred to as Aliquat), Sigma Aldrich or BASF, Antwerp, Belgium) were dried at 80 °C for 16 h before use, unless stated otherwise (Figure 3). Hexyl acetate (99%, Sigma Aldrich, Overijse, BE), γ-butyrolactone (> 99%, Sigma Aldrich, Overijse, Belgium), triethyl phosphate (> 99%, Tokyo Chemical Industry, Zwijndrecht, BE) and Tamisolve^®^ (Tamisolve^®^ NxG, Taminco, Ghent, Belgium) were used as co-solvents of ILs and dried overnight with molecular sieves 3A (Sigma Aldrich, Overijse, Belgium). These sieves were dried in an oven at 70 °C for 45 min, at 300 °C for 4 h and cooled overnight till 180 °C and then kept at this temperature until use. EA (99.5%, Acros Organics, Leuven, Belgium), ethanol (EtOH, 99.99%, Fisher Scientific, Loughborough, UK) and IA (> 97%, Sigma Aldrich, Overijse, Belgium) were applied to make up the feed solution for filtration experiments. Rose Bengal (RB, 1017 Da, Sigma Aldrich, Overijse, Belgium) was applied as test solute when epoxy-cured membranes were synthesized with Aliquat.

### 2.2. TFC Membrane Synthesis

The polymer supports were prepared via NIPS membrane synthesis procedure [5]. Homogeneous polymer solutions of 18 wt% PSf in NMP and 14 wt% PI in NMP/THF (3/1) were made and left overnight for degassing. The polymer solutions were cast on the non-woven substrate with an automatic casting device (Porometer, Belgium) at a constant speed (4.4 × 10^−2^ m·s^−1^) and wet film thickness of 200 µm. For PI films, an evaporation time of 30 s was introduced before immersion in the coagulation bath to allow THF concentration to decrease at the film surface.

The coagulation bath consisted of the monomer MPD (0.1 wt% or 0.3 wt%) next to the non-solvent Milli-Q water, as shown in Figure S2, in order to simultaneously perform NIPS and support impregnation with the first monomer. This synthesis process is here referred to as the simultaneous (SIM) synthesis method [31,32]. In the case of a PI support, HDA (0.5 wt%) was added to the coagulation bath for simultaneous cross-linking of the PI support as well (Figure S3), rendering this membrane preparation method very efficient with respect to use of chemicals, time, and required manipulations. After 5 min in the coagulation bath, the support was removed and further used to perform IP.

Excess aqueous MPD-solution was then removed from the support surface, followed by contacting it with the organic solution containing the second monomer (i.e., TMC). Before the organic solution (here, IL) was added, the IL was vacuum-dried in a Schlenk line (8.8–12.0 mbar) at 80 °C for 16 h, unless stated otherwise. For PA-TFC membranes, a homogeneous TMC solution (1 or 1.5 wt%) in [*C*_4_*mim*][*Tf_2_N*], [*C*_4_*mpyr*][*Tf_2_N*] and Aliquat was prepared, while for epoxy-TFC, the homogeneous solution consisted of BADGE (7 to 13 wt%) in Aliquat. These monomer concentrations in [*C*_4_*mim*][*Tf_2_N*], [*C*_4_*mpyr*][*Tf_2_N*] and Aliquat were chosen, based on membrane performances according to Mariën et al. [18]. These solutions were gently poured on the support and left to react for 60 s (later referred to as reaction time), unless stated otherwise.

After IP, the solution was drained off and the membrane was rinsed with ACN or acetone when a PSf or PI support was used, respectively. The membrane was then dried for 1 min and put in a demineralized water (demi-water) bath for at least 10 min to remove unreacted MPD. Finally, the membranes were stored in demi-water until further use.

### 2.3. Membrane Performance

Membrane filtrations were performed in a high throughput (HT) membrane filtration device which allows simultaneous filtration of 16 membrane coupons with each an active area of 1.77 × 10^−4^ m^2^ [33,34,35]. The pressure driven filtrations in dead-end mode were performed at room temperature (RT) and 20 bar while stirring the feed at 360 rpm to minimize the effect of concentration polarization. The membrane performance was evaluated with (1) a 35 µM RB solution in 20/80 wt% DMF/demi-water for epoxy-cured membranes, and (2) a feed mixture of 12 wt% ethanol, 4000 ppm ethyl acetate (88.105 Da) and 100 ppm isoamyl acetate (130.185 Da) in Milli-Q water for PA-TFC membranes. After adding the feed, the membranes were allowed to stabilize for 1 to 2 h to obtain steady-state. For each membrane, 3 coupons were tested from which the performance was averaged.

Membrane permeance (*P*) was determined through Equation (1) with *V* (L) the permeate volume, *A* (m^2^) the membrane area, *t* (h) the filtration time and Δ*P* (bar) the applied pressure:

(1) $$Permeance=\frac{V}{A\times t\times \Delta P}$$

The retention was calculated by using Equation (2) with *C_f_* and *C_p_* the solute concentration in feed and permeate, respectively:

(2) $$Retention=\left(1-\frac{c_{p}}{c_{f}}\right)\;\times 100\%$$

RB concentrations were determined with a UV-Vis spectrophotometer (UV-1650 PC, Shimadzu) at 554 nm.

The ester and ethanol concentrations were determined by a static headspace gas chromatograph with flame ionization detector (Static HS-GC-FID) (Perkin Elmer Headspace Sampler HS 40). For each vial, 1 g permeate was taken and 100 µm internal standard (IS) was added. The IS used was prepared by diluting 0.05 g propyl propionate with distilled water till 10 g.

Performance comparison of the membranes was done by measuring the permeance of Milli-Q water/EtOH through the membrane and the retention of ethyl acetate (EA) and isoamyl acetate (IA).

### 2.4. Membrane Characterization

The chemical functionality of ILs and membranes was determined through attenuated total reflectance Fourier transform infrared (ATR-FTIR, Bruker, Kontich, Belgium) spectroscopy. The samples were oven-dried before 64 scans were taken at a resolution of 2 cm^−1^ with a Bruker Vertex 70 FTIR spectrometer using a diamond crystal.

Proton nuclear magnetic resonance (^1^H-NMR, Bruker, Kontich, Belgium) spectrometry was used to verify the chemical composition of Aliquat, and to determine whether TMC was hydrolysed and/or esterified in Aliquat or Aliquat/co-solvent system. Each sample was diluted in deuterated chloroform (CDCl_3_) before measurement with the Bruker Avance 300 MHz, taking 64 scans.

Membrane cross-section morphology and top-layer surface were analyzed by scanning electron microscopy (SEM) using a JEOL JSM-6010LV SEM microscope operated at 10 KV. To minimize sample charging in the SEM, the samples were coated with a conductive Au/Pd alloy using a JEOL JFC-1300 Auto Fine Coater.

Transmission electron microscopy (TEM) was used to analyze top-layer cross sections of the membranes at high resolution. Before analysis by a JEOL Atomic Resolution Microscope (ARM200F), equipped with a cold Field Emission Gun (cold-FEG) and probe aberration corrector operated at 200 KV, the membrane samples were embedded in an araldite resin (Polyscience) and cut into ultrathin cross sections of 40 nm using a Reichert Ultracut E microtome.

Thermal properties of the samples were studied using a thermogravimetric analysis (TGA) Q500 from TA Instruments. The thermal degradation process was analyzed by heating approximately 2 mg of sample up to 650  °C at 10  °C/min in N_2_ or O_2_ atmosphere.

Contact angle measurements were performed to analyze the membrane hydrophilicity, using a Krüss DSA 10-Mk2 drop shape analyzer. Droplets of 2 µL were applied and the values of 5 droplets on each sample were averaged.

### 2.5. Solvent Viscosity

Rheological measurements were performed on a stress-controlled rheometer (Anton Paar MCR501) with cone-plate geometries (CP50/1 and CP25/4) and evaporation blocker. The sample temperature was controlled at 23 °C by a Peltier element with accuracy around 0.1 °C. Viscosity as function of shear rate was probed in steady shear flow (from 0.01 to 10 1/s with 5 datapoints per decade for the low-viscosity samples and from 0.001 to 1 1/s with 5 datapoints per decade for the high-viscosity sample). The RheoPlus software (Anton Paar GmbH, Graz, Austria) was used for data acquisition and analysis.

The viscosities were calculated using the derived Stokes–Einstein Equation for highly viscous media (Equation (3)), with *k_b_* the Boltzmann constant (-), *T* the absolute temperature (K), *η* the dynamic viscosity of the solvent (Pa s), *a* the hydrodynamic radius of the solute (m) and *D* the solute diffusion coefficient (m^2^·s^−1^) [18,36,37].

(3) $$D=\;\frac{k_{B}T\;}{4\pi \eta a\;}\;R_{D}=\;\frac{D_{IL}}{D_{\left[C4mim\right]\left[Tf2N\right]}\;}\;$$

Next, for a similar temperature and solute hydrodynamic radius of all ILs, the ratio *R_D_* of solute diffusion coefficients in Aliquat or [C_4_mpyr][Tf_2_N] (*D_IL_*) to that of [C_4_mim][Tf_2_N] (*D*_[C4mim][Tf2N]_) could be defined by Equation (4):

(4) $$R_{D}=\;\frac{D_{IL}}{D_{\left[C4mim\right]\left[Tf2N\right]}\;}\;$$

### 2.6. Vial Tests for Interfacial Polymerization

IP without support was performed in glass vials to visually determine PA and epoxy film formation. First, mixtures with various concentrations of MPD or HDA in Milli-Q water and TMC or BADGE in Aliquat were prepared in separate vials. Thereafter, the Aliquat mixture was poured gently in the glass vial containing the Milli-Q water. The formation of a PA or poly(*β*-alkanolamine) film at the interface was visually determined. IP in absence of a support is further referred to as the “vial test”.

### 2.7. Water Content of Ionic Liquids

The water concentration in the ILs and co-solvents was determined by an automatic Karl Fischer (KF) titration. A sample of 1.5–2 mL was weighed and inserted into the device. For samples with water concentrations above 1.0 × 10^5^ ppm, the Volumetric KF titrator (Mettler Toledo V20S, Zaventem, BE) was used, while for lower water concentrations, the Coulometric KF titrator (Mettler Toledo C30S, Zaventem, Belgium) was used. In the case of dried Aliquat, ACN (<200 ppm H_2_O) was added to the sample to lower the viscosity.

## 3. Results and Discussion

### 3.1. PA-TFC Membrane Synthesis with IL

A previous study by Mariën et al. showed the potential use of ILs (i.e., [*C*_4_*mim*][*Tf_2_N*] and [*C*_4_*mpyr*][*Tf_2_N*]) as organic phase during synthesis of PA-TFC membranes [18,19]. These ILs are known to be relatively costly, and therefore a much less expensive ionic liquid, i.e., Aliquat, was applied now as organic phase for IP (Table S1).

When selecting an IL as organic phase, following important properties of the IL are considered to influence the IP process: (i) water immiscibility to create a biphasic system during IP, (ii) dynamic viscosity (*η*) and surface tension (*σ*) which influence the solubility and diffusivity of the amine monomer in the reaction zone. This zone is known to lie mostly in the organic phase due to the low solubility of acid chlorides in water.

These properties mainly depends on the nature of the anion [19]. For [*C*_4_*mim*][*Tf_2_N*] and [*C*_4_*mpyr*][*Tf_2_N*], the [*Tf_2_N*] anion comprises fluorine groups which are bound to a carbon atom. Aliquat, on the other hand, is composed of a large organic cation (i.e., tri-C8-10-alkylmethylammonium) with a chloride anion (Figure 3). Aliquat is claimed to be water insoluble, but absorption of low concentrations of water from ambient atmosphere is still possible due to its hygroscopic nature.

Before the TFC membrane synthesis method using an IL can be further optimized, the support type and potential use of Aliquat (compared to [*C*_4_*mim*][*Tf_2_N*] and [*C*_4_*mpyr*][*Tf_2_N*]) need to be determined.

#### 3.1.1. Selection of Support

**Figure 4:**
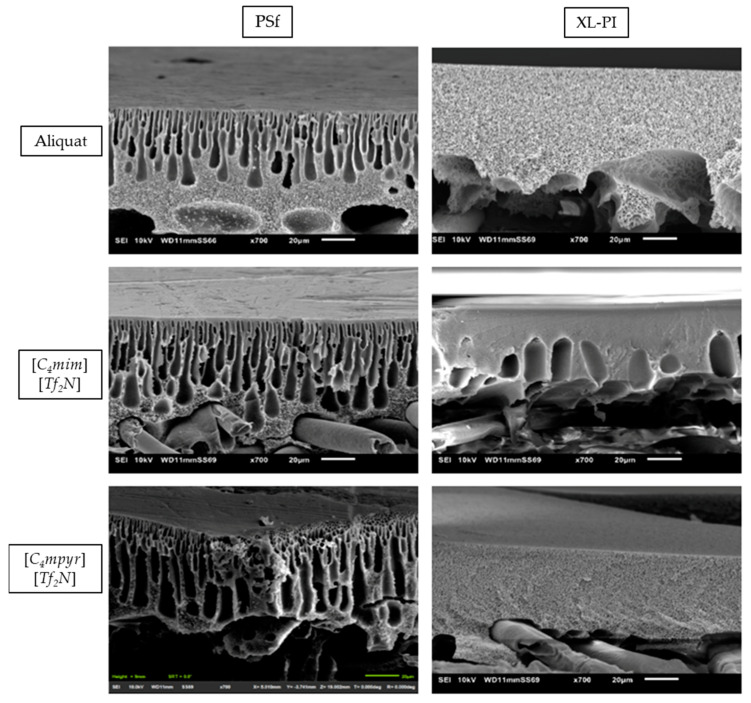
Cross-section scanning electron microscopy (SEM) images of the different membranes synthesized by using different ionic liquids (ILs) on the 2 types of support.

It is known that the top-layer characteristics are influenced by the morphological and chemical properties of the support [6,38,39,40,41,42]. First, a higher hydrophilicity for the cross-linked PI support was observed (XL-PI (70.83° ± 1.82°) > PI (80.37° ± 2.37°) > PSf (90.50° ± 1.54°)), which is known to also affect the top-layer formation [18,31,39,40,41,43,44]. Next, cross-sectional SEM images of TFC membranes using the different ILs as organic phase were shown (Figure 4). The cross-linked PI supports showed significantly less macrovoids compared to the PSf supports.

**Figure 5:**
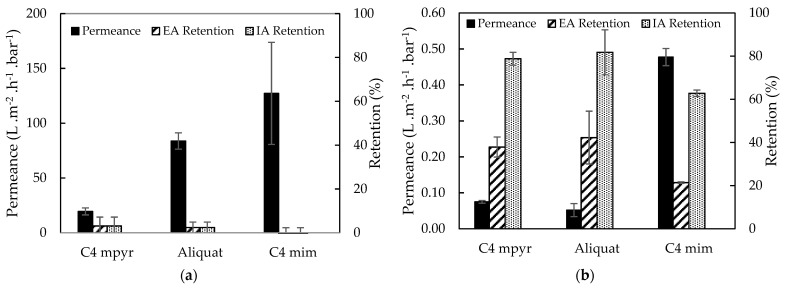
Water/EtOH permeance, EA and IA retentions of TFC membranes synthesized from different ILs as organic phase on (**a**) PSf and (**b**) XL-PI support. With C4 mpyr for [*C*_4_*mpyr*][*Tf_2_N*] and C4 mim for [*C*_4_*mim*][*Tf_2_N*].

The effect of a denser support structure on the membrane performance was observed in Figure 5. As expected, membranes with cross-linked PI supports showed lower water/EtOH permeances and significantly higher EA and IA retentions (3.09 vs. 37.89% of EA retention for [*C*_4_*mpyr*][*Tf_2_N*]) than the membranes with PSf supports. For this reason, cross-linked 14 wt% PI supports were further used during TFC membrane synthesis.

#### 3.1.2. Selection of IL Type

An overview of the different IL properties is given in Table 1, with η the dynamic viscosity, σ_water-organic_ the interfacial tension and *R_D_* the diffusion coefficients ratio.

From Equations (3) and (4), it is clear that a lower diffusivity of MPD was estimated in [*C*_4_*mpyr*][*Tf_2_N*] (*R_D_* = 0.67) and Aliquat (*R_D_* = 0.02) compared to [*C*_4_*mim*][*Tf_2_N*].

Based on these properties, it was thus expected that the diffusion of MPD before reacting with TMC in Aliquat and [*C_4_mpyr*][*Tf_2_N*] is slowed down, resulting in a thinner PA top-layer and higher permeance [18]. Moreover, [*C_4_mpyr*][*Tf_2_N*] and Aliquat have a higher surface tension (σ_water-organic_); hence, a lower partial miscibility of the phases is anticipated, leading to a more limited transport of MPD across the interface [18].

However, the comparison between the performances of TFC membranes prepared on XL-PI supports using the different ILs as organic phase, showed somewhat different results (Figure 5b). In general, a permeance-retention trade-off was observed [48], but lower permeances were observed for [*C*_4_*mpyr*][*Tf_2_N*] and Aliquat compared to [*C*_4_*mim*][*Tf_2_N*]. It was therefore tentatively suggested that due to their higher viscosity, there was still IL present on the membrane surface during the filtration, causing a decrease in permeance [19].

Based on the above characteristics and performances, Aliquat was considered as a suitable, inexpensive alternative IL for the synthesis of TFC membranes. Therefore, Aliquat will be further used to optimize the TFC membrane synthesis.

#### 3.1.3. Reaction Time and Aliquat/Water Content

Due to the hygroscopic character of Aliquat, sorbed water could hydrolyse TMC and decrease its reactivity during IP in Aliquat. The resulting lower degree of cross-linking and changed hydrophilicity of the PA top-layer could therefore negatively influence its filtration performance [18,49,50,51]. However, a decreased reactivity of water in several ILs has been described in literature [18,50,52,53,54]. A possible hypothesis, according to Mariën et al. and Welton et al., is that water forms strong H-bonds with the anions of the ILs, reducing the probability of hydrolysis [19,50]. Mariën et al. confirmed that TFC membranes prepared in [*C*_4_*mim*][*Tf_2_N*] did not result in a decreased retention even for high water contents in the IL [18,19].

In order to control the potential hydrolysis of TMC in ILs, a standard pre-treatment to vacuum-dry Aliquat was set-up. Afterwards, a minimal and maximal Milli-Q water concentration of 1.5 and 21.3 wt% in Aliquat (Ali^1.5^ and Ali^21.3^) was determined. With the increase in water concentration, the associated viscosity of Aliquat decreased to 137 mPa·s.

To determine the PA formation in highly viscous Aliquat samples Ali^1.5^ and Ali^21.3^, concentrations of MPD and TMC were increased to 0.3 w/v% and 1.5 wt% respectively. In addition, the reaction time (Section 2.2) was stepwise increased to 300 s.

**Figure 6:**
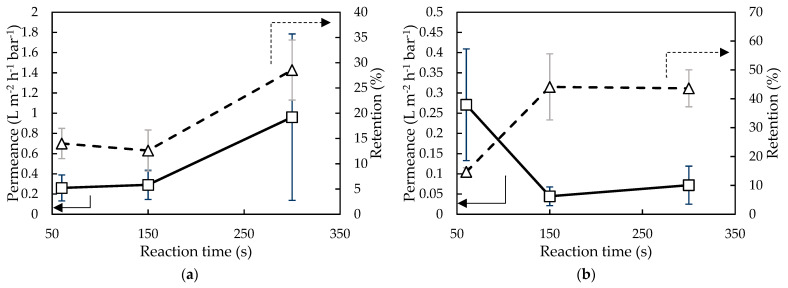
Milli-Q water/EtOH permeance and EA retention of membranes synthesized with (**a**) 1.5 wt% and (**b**) 21.3 wt% H_2_O in Aliquat and with varying reaction times (i.e., 60, 150, and 300 s).

In Figure 6, the effect of reaction time on membrane performance using Ali^1.5^ and Ali^21.3^ is shown. For both membranes synthesized, an increase in retention was observed when increasing the reaction time: 104% from 60 s to 300 s for Ali^1.5^ and 197% for Ali^21.3^. This could indicate that with increasing reaction time, a denser top-layer was obtained which results in a higher retention but lower permeance [18,55].

**Figure 7:**
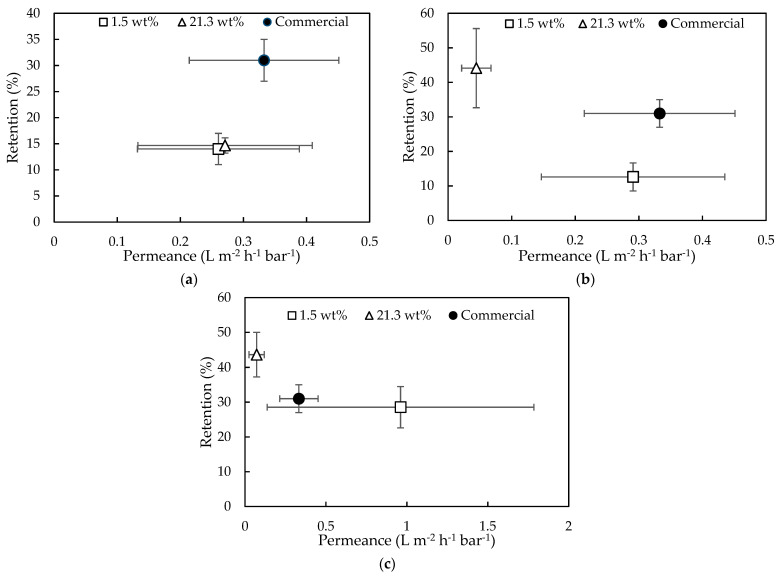
Water/ EtOH permeance and EA retention of GEA RO membrane (type AF) and membranes synthesized with IL containing 1.5 wt% and 21.3 wt% H_2_O, applying (**a**) 60 s, (**b**) 150 s, and (**c**) 300 s reaction time.

Additionally, the performances of these membranes were grouped per reaction time and compared to the performance of the commercial GEA RO membrane (type AF) (Figure 7).

With an increasing reaction time from 60 s to 300 s (Figure 7a–c), the performances of Ali^21.3^ membranes moved towards higher retentions but lower permeances. While for the same increasing reaction times, the Ali^1.5^ membranes obtained comparable retention values but higher permeances. However, no clear trend could be noted due to high standard deviation.

The performances of the synthesized membranes thus approached those of the commercial membrane, especially when a reaction time of 300 s was applied.

Following optimization steps were performed with Aliquat from BASF (Germany) instead of Sigma Aldrich (used in previous experiments) as the latter was discontinued. The chemical composition of both was the same, but a new maximal water concentration of 17.5 wt% was determined. The reproducibility of previous data was confirmed by observing similar membrane performances using Aliquat from BASF (Figures S4 and S5).

It is suggested that membranes prepared from Aliquat with intermediate water content could exist with still better performance than the commercial membrane. However, an intermediate water concentration of 9.5 wt% resulted in an increased retention but decreased permeance compared to the commercial membrane (Figure S5).

Since membranes prepared from an IL with 17.5 wt% H_2_O (Ali^17.5^) showed the highest retentions and smaller standard deviations, this composition will be further used to optimize the monomer concentrations for a suitable PA film formation.

#### 3.1.4. Determination of the Monomer Concentration

Ionic liquid properties such as viscosity and surface tension are known to affect the optimal monomer concentrations of MPD and TMC for the formation of a suitable cross-linked PA film during IP [18,19,54]. Therefore, the monomer solubility limits, within which PA film formation is possible, were determined via vial tests.

The concentrations used in the vial tests ranged from 0.1 to 2.0 w/v% and 0.1 to 4.0 wt% for MPD and TMC, respectively. These concentrations were based on: (i) the maximal solubility of TMC in Aliquat and MPD in MilliQ water (7 wt% and 31.5 wt%, respectively), (ii) the concentrations used in the conventional system (0.1 w/v% TMC and 2.0 w/v% MPD), and (iii) economic cost.

As shown in Table 2, no or very little PA film formation occurred. With increasing concentrations of both monomers, a “cloudy fragile white layer” or insufficient cross-linked polymer was observed within a few seconds reaction time (Figure S6a,b). For comparison, two vials with the conventional system (i.e., hexane as organic phase) were prepared (Figure S6c,d). In both vials, a highly cross-linked PA film was obtained after 30 s.

**Figure 8:**
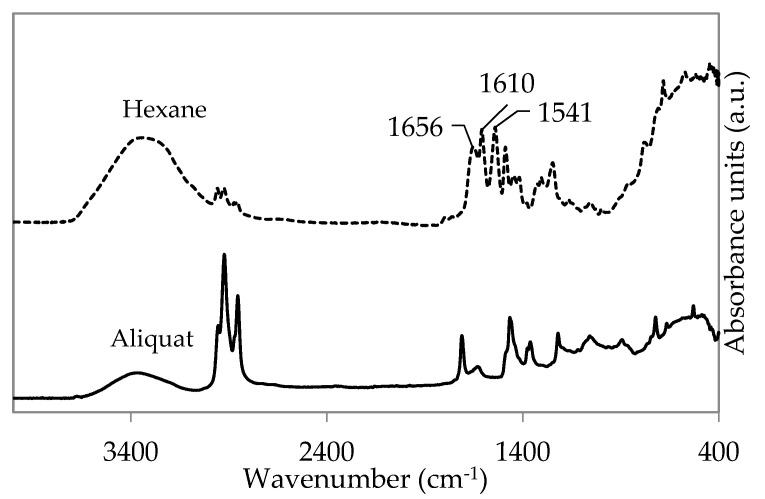
Attenuated total reflectance Fourier transform infrared (ATR-FTIR) spectra of PA film prepared using 1.5 wt% TMC in both hexane (dotted line) and Aliquat (full line).

ATR-FTIR spectroscopy of the “cloudy white layer” was used to determine whether PA film formation occurred using H_2_O/Aliquat mixtures (Figure 8). No characteristic amide absorption bands (at 1656, 1610, and 1541 cm^−1^) could be distinguished, supporting the hypothesis that no well cross-linked PA film was formed during the vial test. This absence of PA-formation could indicate that TMC was partly or fully hydrolysed in the H_2_O/Aliquat mixture.

#### 3.1.5. Stability of TMC in Aliquat

To determine the stability of TMC in Aliquat mixtures, ATR-FTIR and ^1^H-NMR spectra of vacuum-dried (i.e., at 80 °C for 16 h) and non-dried Aliquat samples with and without TMC added were analyzed.

**Figure 9:**
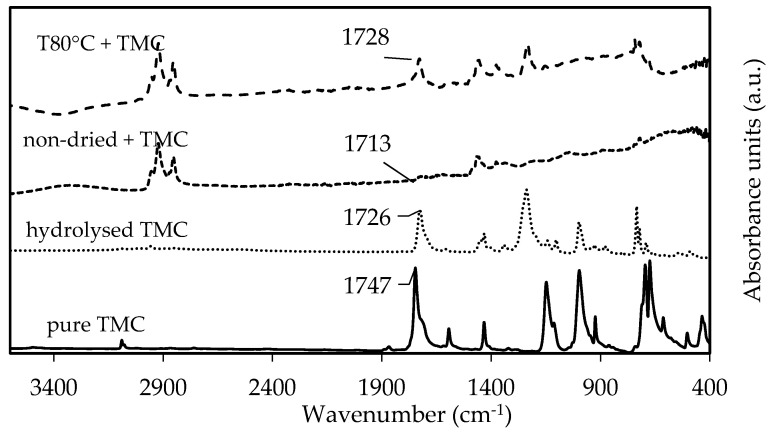
ATR-FTIR spectra of 1.5 wt% pure TMC (full line), hydrolyzed TMC (striped line), TMC in non-dried (dotted line), and vacuum-dried Aliquat at 80 °C (striped line).

As expected, no chemical changes in Aliquat were observed during vacuum-drying (Figure S7). Moreover, the decrease in water concentration upon drying was confirmed by Karl Fischer titration with 2000 ppm and 11,000 ppm for vacuum-dried Aliquat and non-dried Aliquat, respectively. When 1.5 wt% TMC was added to vacuum-dried and non-dried Aliquat, the ATR-FTIR spectra in Figure 9 showed a C=O stretching peak of TMC in both samples (1728 cm^−1^ and 1713 cm^−1^, respectively). Comparing it to the ATR-FTIR spectra of pure and hydrolysed TMC (peak at 1747 cm^−1^ corresponds to the C=O stretching peak of the unreacted acyl chloride of TMC, while 1726 cm^−1^ corresponds to the C=O stretching peak of carboxylic acid due to hydrolysis of TMC [6,56]), this indicates that the peaks between 1710 and 1730 cm^−1^ were due to C=O stretching of carboxylic acid from hydrolysed TMC. This C=O stretching peak of carboxylic acid was suggested to shift due to hydrogen-bonding with water [57].

On the other hand, due to the presence of octanol and decanol in Aliquat (Figure S8), it was suggested that an additional esterification reaction of TMC could occur (Figure S9). Therefore, it was suggested that the peaks of 1713 cm^−1^ and 1728 cm^−1^ in Figure 9 could also correspond to C=O stretching peaks of aryl esters since they appear between 1715 and 1730 cm^−1^ [56]. Hence, both hydrolysis and esterification of TMC could negatively influence the PA reaction. To confirm which reaction occurs (i.e., hydrolysis and/or esterification), ^1^H-NMR analysis was performed on the same samples.

No significant changes in chemical composition of Aliquat were observed upon vacuum drying, as expected (Figure S10a). After adding 1.5 wt% TMC, a chemical shift (δ) at 4.3 ppm was observed, which corresponds to protons next to esters (Figure S10b) [56]. This confirmed that an additional esterification of TMC occurred in Aliquat when TMC was added. From close-ups Figure S10c,d, the chemical shift at 11.8 ppm originated from protons of carboxylic acids, which indicated the expected hydrolysis reaction with TMC. The protons in the benzene ring resulted in a multiplet at 8.8 ppm, which indicated that different functional groups (i.e., acyl chloride, carboxyl group, and/or different ester groups) were present on the benzene ring [56]. Hence, the ^1^H-NMR spectra confirmed that both esterification and hydrolysis of TMC occurred simultaneously. These results support why no highly cross-linked PA film could be obtained.

#### 3.1.6. Use of Co-Solvents

To minimize the effect of hydrolysis and/or esterification, the concentration of water and alcohol in Aliquat was further decreased via vacuum-drying at higher temperature for longer time (i.e., 100 °C for 48 h). Additionally, co-solvents instead of water were used.

The boiling points of water, octanol, and decanol at vacuum are 5.2–9.4 °C, 93.9–98.9 °C, and 132.1–137.4 °C, respectively. Hence, to minimize vaporization of trioctylmethylammonium chloride, Aliquat was vacuum-dried at 100 °C for 48 h. As expected, no significant structural changes in Aliquat could be observed when increasing the temperature from 80 °C to 100 °C (Figure S11).

Instead of adding 17.5 wt% water, 17.5 wt% of co-solvents was now used to improve the transport and interfacial properties [58,59]. The co-solvents used were acetone, acetronitrille, Tamisolve^®^, 𝛾-butyrolactone, hexyl acetate, and triethyl phosphate (structures in Figure S12). These solvents were chosen for their low toxicity and viscosity [58,59]. The final water concentrations of the different dried co-solvents and Aliquat/co-solvent mixtures were determined by Karl Fisher titrations (Table S2). The mixtures with Tamisolve^®^, acetonitrile and 𝛾-butyrolactone) were below 1000 ppm. In theory, not enough water was present to hydrolyse all TMC molecules. Water concentration of the Aliquat/acetone mixture could not be correctly measured by the Karl Fischer titration, as acetone is known to be reacting with methanol in the Karl Fischer reaction medium [60].

The vial test was then repeated for Aliquat/co-solvent mixtures with 1.5 wt% TMC combined with 0.3 w/v% MPD in Milli-Q water. A similar “cloudy” structure was observed during vial tests but less pronounced (Figure S13). Additional ATR-FTIR measurements again indicated hydrolysis and/or esterification upon addition of 1.5 wt% TMC by observing an absorption band at 1728 cm^−1^ (Figure S14). No significant absorption band of TMC could be observed for acetone and hexylacetate, probably due to peak overlap with the C=O stretch of the co-solvent

Additional evidence of hydrolysis and/or esterification (due to presence of alcohols) of TMC in Aliquat was provided by the ATR-FTIR spectrum of 1.5 wt% TMC in (pure) vacuum-dried Aliquat at 100 °C (Figure S15) and supported by ^1^H-NMR spectra (Figures S16 and S17). It was suggested that hydrolysis and esterification of TMC occurred much faster in Aliquat than in other ILs due to the presence of tri-(C8-10)-amine. The tertiary amines could act as catalyst and accelerate hydrolysis and esterification of TMC (Figure S18) [61,62]. Based on the fact that TMC was not submitted to hydrolysis in an overload of water while stirring for 1 h (ATR-FTIR spectra given in Figure S19) but did hydrolyse in Aliquat after stirring for 30 min to 1 h, the hypothesis stated above of hydrolysis and esterification in Aliquat was confirmed.

#### 3.1.7. Influence of Aliquat as Organic Phase on Membrane Performance

To determine the influence of Aliquat on the membrane performances in Section 3.1.1 and Section 3.1.3, additional filtrations using a XL-PI support were performed. The permeances of membranes obtained with (1) non-dried Aliquat, (2) non-dried Aliquat + 1.5 wt% TMC, (3) Ali^17.5^, and (4) Ali^17.5^ + 1.5 wt% TMC showed no significant differences due to high standard deviations (i.e., 0.14 ± 0.06 L·m^−2^·h^−1^·bar^−1^, 0.14 ± 0.05 L·m^−2^·h^−1^·bar^−1^, 0.15 ± 0.06 L·m^−2^·h^−1^·bar^−1^, and 0.08 ± 0.06 L·m^−2^·h^−1^·bar^−1^, respectively). Compared to the performances of cross-linked and non-cross-linked PI supports (307.13 ± 30.61 L·m^−2^·h^−1^·bar^−1^ and 424.22 ± 80.24 L·m^−2^·h^−1^·bar^−1^, respectively), the permeances of the membranes prepared with Aliquat and H_2_O/Aliquat mixture was significantly lower. Moreover, a significant increase in retentions of these membranes was observed (e.g., 0.10 ± 0.02% to 8.17 ± 4.12% when Ali^17.5^ was applied). Next, addition of TMC did not result in a significant effect on the membrane performances.

Based on these results, results from Section 3.1.1 and Section 3.1.3 and an indicative filtration test with 35 µM RB (Figure S20), it was suggested that the viscosity of Aliquat plays a significant role in the finale performance of the membrane. The lower the viscosity of Aliquat (i.e., diluted with Milli-Q water), the more readily it could intrude in the support. Thus, a more homogeneous spread over the membrane surface together with deeper pore filling can be expected. This explains the observed higher membrane retentions with increasing water concentration in Figure 7 and with increasing reaction time in Section 3.1.3 [63,64,65]. This could also explain the generally observed high standard deviations for membranes prepared from IL with low water content (i.e., more viscous).

From SEM images of the membrane surface in Figure S22, the typical “ridge and valley” structure of a conventional PA top-layer could not be observed [18,19]. Such top-layer morphology is possible when ILs were used instead of hexane according to Mariën H. et al. [18,19].

### 3.2. Epoxy-Curing with Aliquat

**Figure 10:**
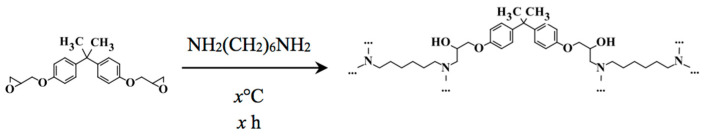
Reaction of Bisphenol A diglycidyl ether (BADGE) and 1,6-hexanediamine (HDA) with the formation of poly(*β*-alkanolamine) [22].

Since as-received Aliquat contains tertiary amines and alcohols as impurities, these compounds could potentially act as initiators during epoxy-curing. Phenolic glycidyl ethers are cheap, readily available and cure at room temperature. For IP, the epoxide is dissolved in the organic phase and the hardener in the aqueous phase. The epoxy can react with the hardener in several ways, e.g., via nucleophilic ring opening polymerization (NROP) (Figure 10) or anionic ring opening polymerization (AROP, also called homopolymerization) (Figure S23) [22,24].

#### 3.2.1. Preliminary Screening

The NROP reaction mechanism led to formation of a poly(*β*-alkanolamine) with BADGE as epoxide monomer in the organic phase (i.e., Aliquat) and HDA as hardener in the aqueous phase (Figure 10) [22].

Three vials were prepared with different monomer concentrations (Table S3). After a prolonged reaction time of one month, no film formation was observed. However, a subsequent acceleration of the polymerization process at 80 °C for one additional month resulted in a film formation in vial (3) (Figure S24a). This was expected since curing times at RT can vary from seconds to months or even years. The successful epoxy curing was confirmed by ATR-FTIR of the isolated film, as given in Figure S24b.

The disappearance of the epoxy absorption bands at 862 and 915 cm^−1^ (i.e., COC and CO stretching of the oxirane ring) indicated that the epoxy ring opening had occurred [66]. Further, the appearance of strong absorption bands between 1105 and 1083 cm^−1^ indicated the presence of a secondary alcohol (CO stretching) and a tertiary amine (CN stretching) [47,56]. This indicated that epoxy-curing had occurred, and a poly(*β*-alkanolamine) (Figure S25) film was formed. It should be noted that the tertiary amine absorption band might also be caused by traces present in Aliquat.

Moreover, no to negligible hardening of the Aliquat bulk solution was observed after preparation of a homogeneous solution with BADGE. It is suggested that the tertiary amines present in Aliquat (tri-(C8-C10)-amine) were too bulky to act as good initiators.

#### 3.2.2. Influence of Monomer Type and Synthesis Conditions

Based on the preliminary screenings, a more practical- and time-efficient approach was needed. Therefore, two types of phenolic glycidyl ether monomers were studied (i.e., BADGE and EPON^TM^ SU-8, further referred to as E2 and E8) in combination with different temperatures (i.e., RT vs. 80 °C) and a maximal reaction time of 80 h.

Vial tests were performed for each monomer type, whereby solutions of various HDA concentrations in demineralized water were brought in contact with the same concentrations for E2 or E8 in Aliquat (Table 3). A lower concentration for E8 was used, as a more efficient epoxy-curing was expected due to the eight reactive sites instead of two for E2. Additional images of these vial tests are shown in Tables S4 and S5. A more successful epoxy-curing for E2 was observed starting from a HDA and E2 concentration of 10 wt%. While for E8 only cloudy film formation could be observed with 4 wt% HDA/E8 concentration. Higher concentrations of both HDA and E8 resulted in incomplete dissolution, limiting the use of E8 as potential alternative monomer. As expected, a longer reaction time (i.e., from 20 to 80 h) resulted in a thicker film, while increasing the temperature for a same reaction time (i.e., RT vs. 80 °C) resulted in more rapid polymerization. Since successful isolation of a poly(*β*-alkanolamine) film at RT for a reaction time of 20 h could be observed, this was considered a very interesting new method to obtain scalable epoxy-TFC-membranes.

**Figure 11:**
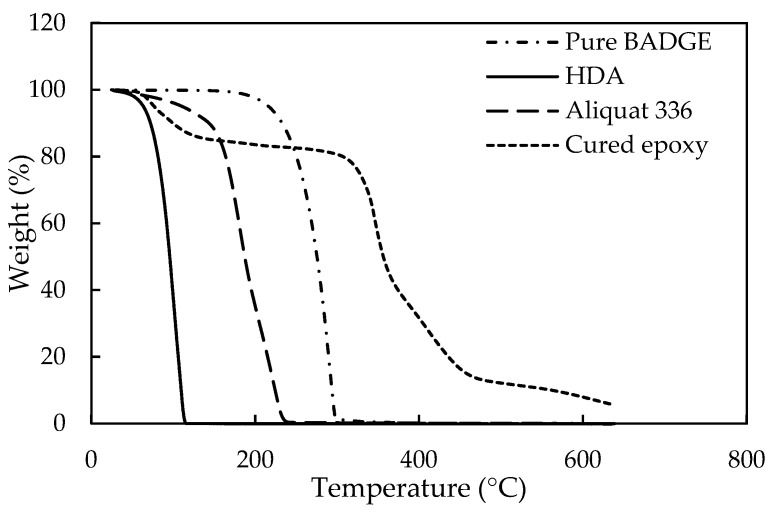
Thermogravimetric analysis (TGA) measurements of pure HDA (full line), pure Aliquat (striped line), pure E2 (striped/dotted line) and cured epoxy (dotted line).

Additional evidence for a successful epoxy-curing of E2 during IP, was provided by comparing the ATR-FTIR images of the obtained poly(*β*-alkanolamine) film with literature data [66], as given in Figure S26. No significant differences between the spectra could be observed, confirming successful curing. Curing of E2 was further characterized through TGA in N_2_ atmosphere (Figure 11). The film showed a relatively good thermal stability. A first degradation (at ~100 °C) was attributed to unreacted HDA and, subsequent degradation (at ~350 °C) to the E2/Aliquat mixture. The final degradation (at > 400 °C) was an indication of cured E2 structures or even the formation of poly(*β*-alkanolamine) bonds.

#### 3.2.3. Epoxy-TFC Membrane Synthesis

Based on above film formation tests, the SIM synthesis method was applied to obtain an epoxy-cured TFC membrane. Here, monomer concentrations of 10 and 13 wt% for HDA and BADGE were used. The membrane synthesis was performed at RT and with an IP-reaction time from 24 h to 72 h.

Evidence for the successful curing of E2 (and thus a poly(*β*-alkanolamine) film formation) on top of the cross-linked PI support, was provided by ATR-FTIR spectroscopy (Figure S27). As expected, three characteristic amide peaks appeared (at 1654, 1601, and 1540 cm^−1^) next to the non-woven related peaks when 10 or 13 wt% HDA was added to the coagulation bath for PI cross-linking. When 10 or 13 wt% E2 was added to induce IP, the peaks of cross-linked PI decreased and characteristic peaks of the epoxy structure at 1034 cm^−1^ (i.e., C–O–C symmetric stretching of Ph–O–C group) appeared. Again, the disappearance of epoxy peaks at 863 and 911 cm^−1^ indicated that epoxy ring opening occurred.

#### 3.2.4. Epoxy-TFC Membrane Performance

**Figure 12:**
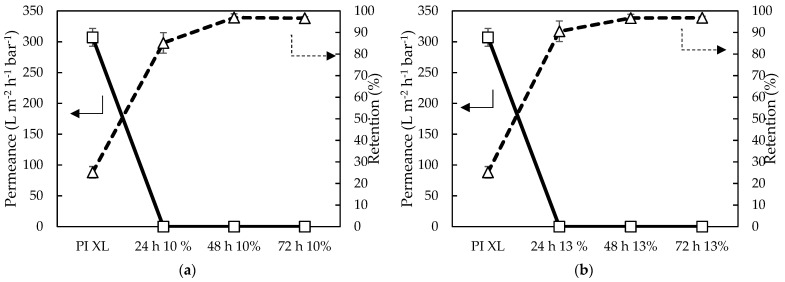
Filtration performance of (**a**) 10 wt% HDA in water with 10 wt% E2 in Aliquat and (**b**) 13 wt% HDA with 13 wt% E2 using a high throughput (HT) membrane filtration for simultaneous screening of 16 membranes at 20 bar and room temperature (RT).

From filtration with 35 µM RB in a 20/80 wt% DMF/H_2_O feed, a similar trend was observed (Figure 12): longer reaction times resulted in higher RB retentions. A plateau was reached from 48 h onwards, indicating that 48 h was sufficient to obtain a performing epoxy-TFC membrane. However, a very low permeance for all membrane samples was observed, i.e., < 0.01 L·m^−2^·h^−1^·bar^−1^, making these membranes impractical for use in membrane applications. This could be explained by the fact that very high concentrations of both monomers were used.

## 4. Conclusions

Inexpensive Aliquat was tested as organic phase to render IL-based IP more economical for upscaling. No suitable cross-linked PA film was formed, even after careful optimization of several synthesis parameters: (i) pretreatment of Aliquat by removing water and alcohols via vacuum drying and dissolving it in dried co-solvents, (ii) optimized monomer concentration in aqueous and organic phase via vial tests, (iii) prolonged reaction time due to higher viscosity, (iii) control of the water concentration in Aliquat. Nonetheless, hydrolysis and esterification of TMC kept occurring. It was therefore concluded that a high concentration of alcohols (initially present in this low grade industrial product) remained present in the treated Aliquat and that the present tertiary amines can act as catalysts for the hydrolysis and esterification reactions.

The unexpected decrease in H_2_O/EtOH permeance and increase in EA retention was found to be caused by a layer of Aliquat on the support surface.

A successful proof of concept for the use of Aliquat as organic phase was delivered for a new type of IP chemistry, involving epoxide-curing of BADGE with HDA. A poly(β-alkanolamine) film layer was obtained during IP with 10 wt% HDA and 10 wt% BADGE. ATR-FTIR and filtration experiments supported the formation of an epoxy-cured top-layer. However, further study is needed to improve the filtration performance by optimizing the monomer concentrations and other synthesis parameters.

## Figures and Tables

**Table 1:** Properties of [*C*_4_*mim*][*Tf_2_N*] and [*C_4_mpyr*][*Tf_2_N*] at 25 °C and Aliquat at 23 °C [45,46,47].

	[*C*_4_*mim*][*Tf_2_N*]	[*C*_4_*mpyr*][*Tf_2_N*]	Aliquat
*η* (mPa·s)	51	76	2941
σ_water-organic_ (mN·m^−1^)	13	34	28
*R_D_* (-) *	1	0.67	0.02

* with *R_D_* the ratio of solute diffusion coefficients in Aliquat or [*C_4_mpyr*][*Tf_2_N*] (*D_IL_*) to that of [*C*_4_*mim*][*Tf_2_N*] (*D*_[C4mim][Tf2N]_).

**Table 2:** Visual determination of PA film formation in vial tests for various concentrations combinations of MPD in water and TMC in Aliquat.

MPD (w/v%) →	0.1	0.3	0.5	1.0	2.0
TMC (wt%) ↓					
0.1	-	-	-	-	±
0.5	±	±	±	±	±
1.5	±	±	±	±	±
2.5	±	±	±	±	±
4	±	±	±	±	±

**Table 3:** Visual determination of epoxy-curing during interfacial polymerization (IP) for various concentrations of (i) HDA (i.e., 4–16 wt%) in water combined with E2 (i.e., 4–16 wt%) in Aliquat, and (ii) HDA (i.e., 1–10 wt%) in water combined with E8 (1–10 wt%)^.^^1^.

	20 h		80 h			20 h		80 h	
HDA (wt%)/E2 (wt%)	RT	T_80 °C_	RT	T_80 °C_	HDA (wt%)/E8 (wt%)	RT	T_80 °C_	RT	T_80 °C_
4/4	-	-	-	-	1/1	-	-	-	-
7/7	-	-	±	±	2/2	-	-	-	-
10/10	±	++	++	++	4/4	±	±	±	±
13/13	+	++	++	++	8/8	NA	NA	NA	NA
16/16	+	++	++	++	10/10	NA	NA	NA	NA

^1^ ‘-’ no film formation, ‘±’ partly cured film, ‘+’ well cured film and ‘++’ well cured thick film.

## Supplementary Materials

The following are available online at https://www.mdpi.com/article/10.3390/membranes11040297/s1: Figure S1: Structure of (a) BADGE and (b) EPON^TM^ Resin SU-8. Figure S2: The traditional process compared to the SIM method for the synthesis of cross-linked TFC membranes based on a PI support. For PSf, cross-linking is not included (adapted from [8,31]). Figure S3: Reaction to cross-link PI with HDA (adapted from [14,33]). Figure S4: Milli-Q water/EtOH permeance and EA retention of membranes synthesized with 17.5 wt% H_2_O in Aliquat. Figure S5: Water/EtOH permeance and EA retention of membranes synthesized with 300 s reaction time and different water concentrations in Aliquat (BASF). Figure S6: Visual determination of creation of PA film using (a) 0.1 wt% TMC in 17.5 wt% H_2_O mixture with 0.3 w/v% MPD in MilliQ water, (b) 4.0 wt% TMC with 1.0 w/v% MPD in MilliQ water after 3 min reaction time, (c) 0.1 wt% TMC in hexane and 2.0 w/v% MPD in MilliQ water, and (d) 1.5 wt% TMC and 0.3 w/v% MPD after less than 1 min reaction time. Figure S7: ATR-FTIR spectra of non-dried (full line), and vacuum-dried Aliquat at 80 °C (dotted line). Figure S8: Chemical composition of all components present in Aliquat 336, as received from the supplier (BASF). Figure S9: (a) hydrolysis of TMC with water, (b) esterification of TMC with octanol and (c) decanol. Figure S10: 300 MHz 1H-NMR spectra of (a) non-dried Aliquat (blue) and vacuum-dried at 80 °C (red), (b) 1.5 wt% TMC in dried (red) and non-dried (blue) Aliquat, (c) a close up of 1.5 wt% TMC in dried Aliquat and (d) a close up of 1.5 wt% TMC in non-dried Aliquat, in CDCl3. On the x-axis, the chemical shift (ppm) is displayed. Figure S11: ATR-FTIR of Aliquat from bottle (full line), vacuum-dried at 80 °C (dotted line) and at 100 °C (striped line). Figure S12: Structures of co-solvents. Figure S13: Determination of cloudy structure when using 0.3 w/v% MPD in MilliQ water with 1.5 wt% TMC in (a) γ-butyrolacton, and (b) acetonitrile as co-solvents. Figure S14: ATR-FTIR spectra of dried Aliquat (100 °C) when 17.5 wt% co-solvents and/or 1.5 wt% TMC was added. ATR-FTIR spectra of pure co-solvent (full line), Aliquat (Al) + co-solvent (dotted line) and Aliquat + co-solvent + TMC (striped line) for co-solvents (a) acetone, (b) acetonitrile (ACN), (c) 𝛾-butyrolactone (𝛾-but), (d) hexyl acetate (hexyl), (e) Tamisolve^®^ (Tami) and (f) triethyl phosphate (triethyl). Figure S15: ATR FTIR spectrum of vacuum-dried Aliquat at 100 °C when 1.5 wt% TMC was added. An absorption band was observer at 1728 cm^−1^, indicating hydrolysis and/or esterification of TMC. Figure S16: The 300 MHz ^1^H-NMR spectra of vacuum dried Aliquat 336 at 100 °C with 1.5 wt% TMC. On the x-axis, the chemical shift (in ppm) is displayed. Figure S17: The 300 MHz ^1^H-NMR spectra of 1.5 wt% TMC in vacuum dried Aliquat 336 at 100 °C for 48 h with 17.5 wt% 𝛾-butyrolactone (green), acetonitrile (red) and Tamisolve^®^ (blue). Figure S18: Possible reaction mechanisms of catalyzed hydrolysis (R’ = H) and esterification (R’ = alkyl) of TMC. Figure S19: ATR-FTIR spectra of TMC in H_2_O stirred for 1 h. Figure S20: RB retentions of membranes with PI support, XL-PI support, Aliquat and Aliquat + 1.5 wt% TMC on XL-PI support. Figure S21: Structure of Rose Bengal. Figure S22: Surface SEM images of (a) typical ridge-and-valley morphology of an aromatic PA top-layer obtained with the conventional system (i.e., with hexane), (b) 1.51 wt% H_2_O and (c) 21.3 wt% H_2_O in Aliquat. Figure S23: Anionic ring opening polymerization of EPON with TMHD. Figure S24: (a) Successful isolation of a poly(β-alkanolamine) film. (b) ATR-FTIR spectra of isolated poly(β-alkanolamine) film (dotted line) and pure BADGE (full line). Figure S25: Structure of poly(β-alkanolamine). Figure S26: ATR-FTIR spectrum of the obtained poly(β-alkanolamine) film (full line) and literature data (dotted line). Figure S27: ATR-FTIR spectra for (a) 10 wt% HDA in water with 10 wt% BADGE in Aliquat and (b) 13 wt% HDA with 13 wt% E2. Table S1: Prices of ILs. Table S2: Water content in dried co-solvents and Aliqaut/co-solvent mixtures. Table S3: Preliminary screening of poly(β-alkanolamine) film formation for three different concentrations of HDA and BADGE. Table S4: Vial tests for different concentrations of E2 and HDA. Table S5: Vial tests for different concentrations of E8 and HDA.
