# Supplementary material for: Use of Ionic Liquids and Co-Solvents for Synthesis of Thin-Film Composite Membranes
Source: Membranes (Basel). 2021 Apr 20;11(4):297. doi: 10.3390/membranes11040297 (PMC8073406; doi:10.3390/membranes11040297)
Supplement: Supplementary file 1 [file membranes-11-00297-s001.zip › membranes-1115113-supplementary.pdf]

Supporting Information: Use of Ionic Liquids and Co-Solvents for Synthesis of Thin-Film Composite Membranes

Peter-Renaat Van den Mooter *, Liridona Dedvukaj and Ivo F. J. Vankelecom *

Membrane Technology Group (MTG)—Division cMACS—Faculty of Bioscience Engineering KU Leuven, Celestijnenlaan 200F, Box 2454, 3001 Leuven, Belgium; peter.vandenmooter1@kuleuven.be
*Correspondence: peter.vandenmooter@kuleuven.be (P.-R.VdM.); ivo.vankelecom@kuleuven.be (I.F.J.V.); Tel.: +32-16329207 (P.-R.V.d.M.); +32-16321594 (I.F.J.V.)

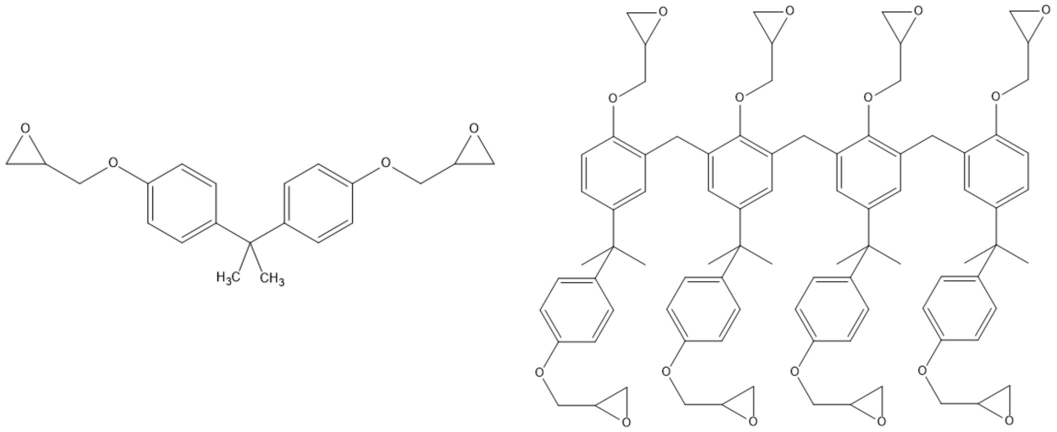

(a) (b)

Figure S1. Structure of (a) BADGE and (b) EPON™ Resin SU-8.

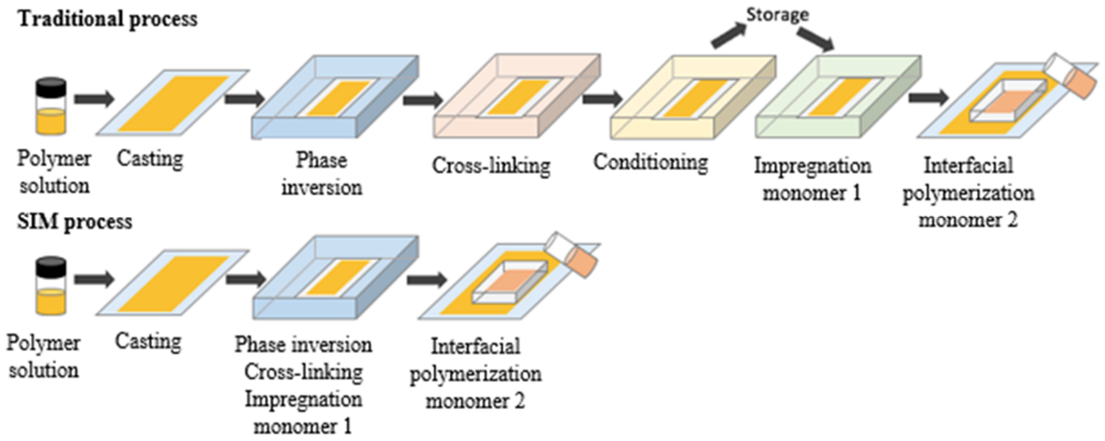

Figure S2. The traditional process compared to the SIM method for the synthesis of cross-linked TFC membranes based on a PI support. For PSU, cross-linking is not included (adapted from [1,2])

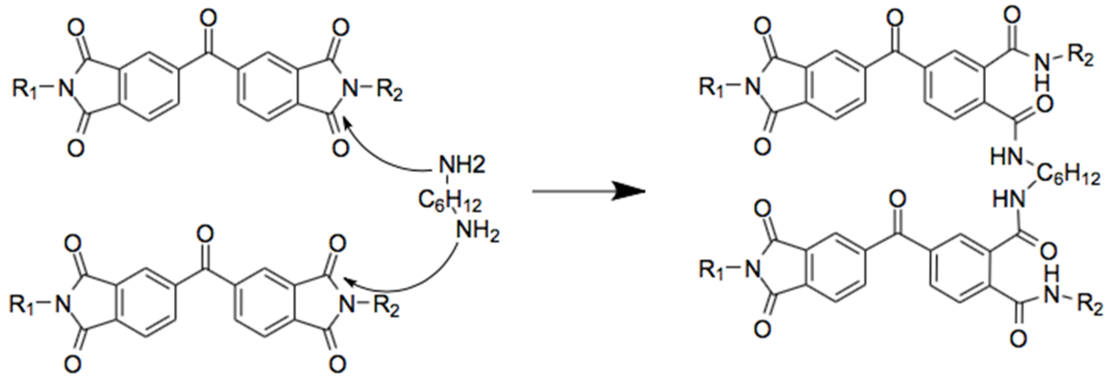

Figure S3. Reaction to cross-link PI with HDA (adapted from [3,4]).

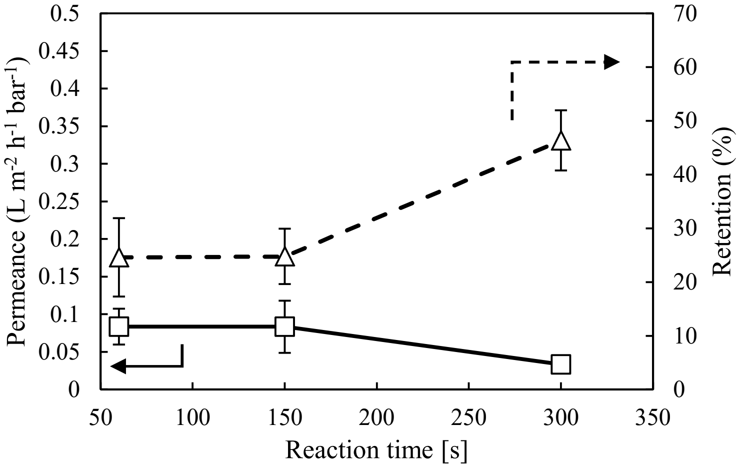

Figure S4. Milli-Q water/EtOH permeance and EA retention of membranes synthesized with 17.5 wt% H_2O in Aliquat.

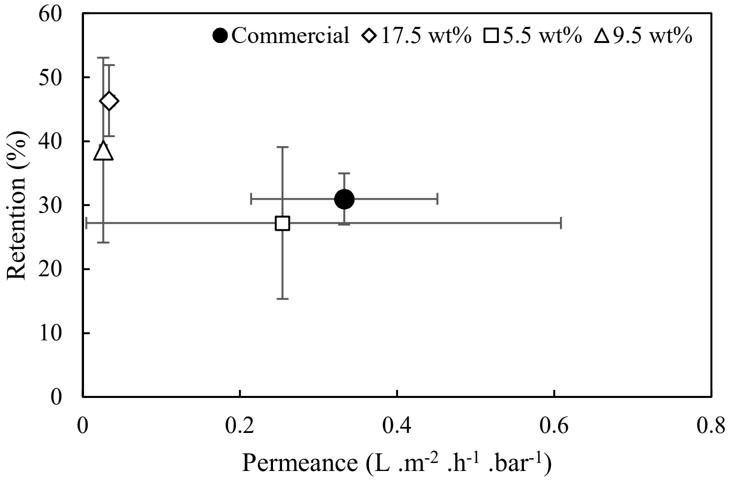

Figure S5. Water/EtOH permeance and EA retention of membranes synthesized with 300 s reaction time and different water concentrations in Aliquat (BASF).

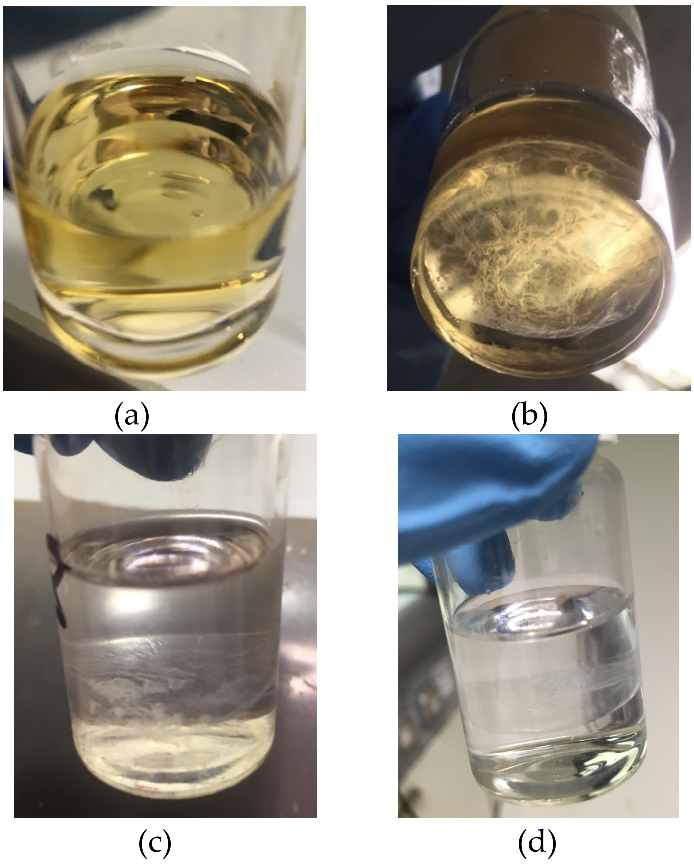

Figure S6. Visual determination of creation of PA film using (a) 0.1 wt% TMC in 17.5 wt% H2O mixture with 0.3 w/v% MPD in MilliQ water, (b) 4.0 wt% TMC with 1.0 w/v% MPD in MilliQ water after 3 min reaction time, (c) 0.1 wt% TMC in hexane and 2.0 w/v% MPD in MilliQ water, and (d) 1.5 wt% TMC and 0.3 w/v% MPD after less than 1 min reaction time.

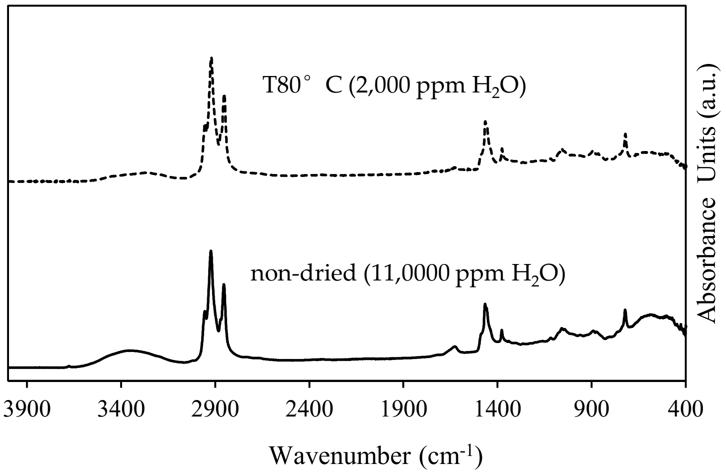

Figure S7. ATR-FTIR spectra of non-dried (full line), and vacuum-dried Aliquat at 80 °C (dotted line).

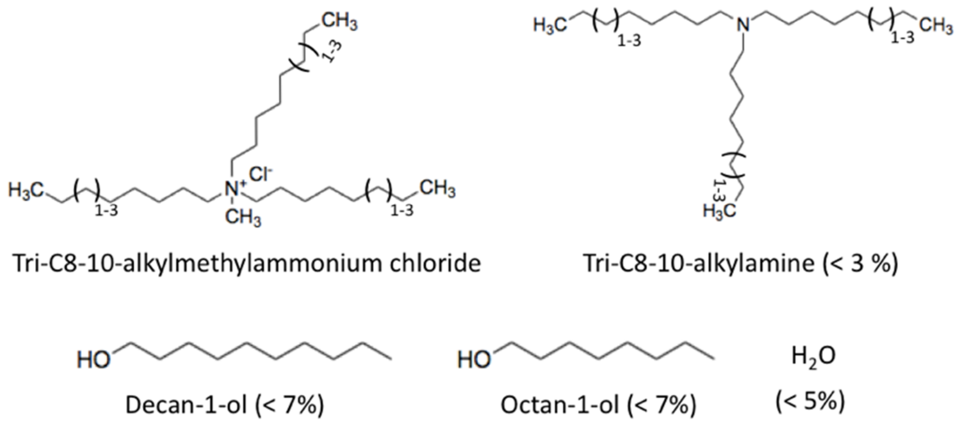

Figure S8. Chemical composition of all components present in Aliquat 336, as received from the supplier (BASF).

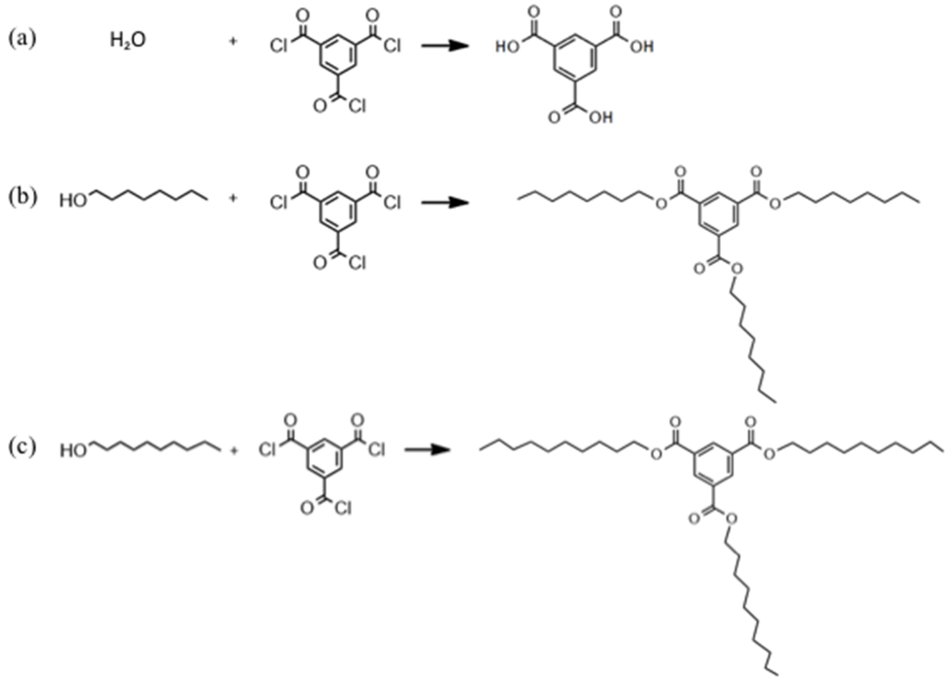

Figure S9. (a) hydrolysis of TMC with water, (b) esterification of TMC with octanol and (c) decanol.

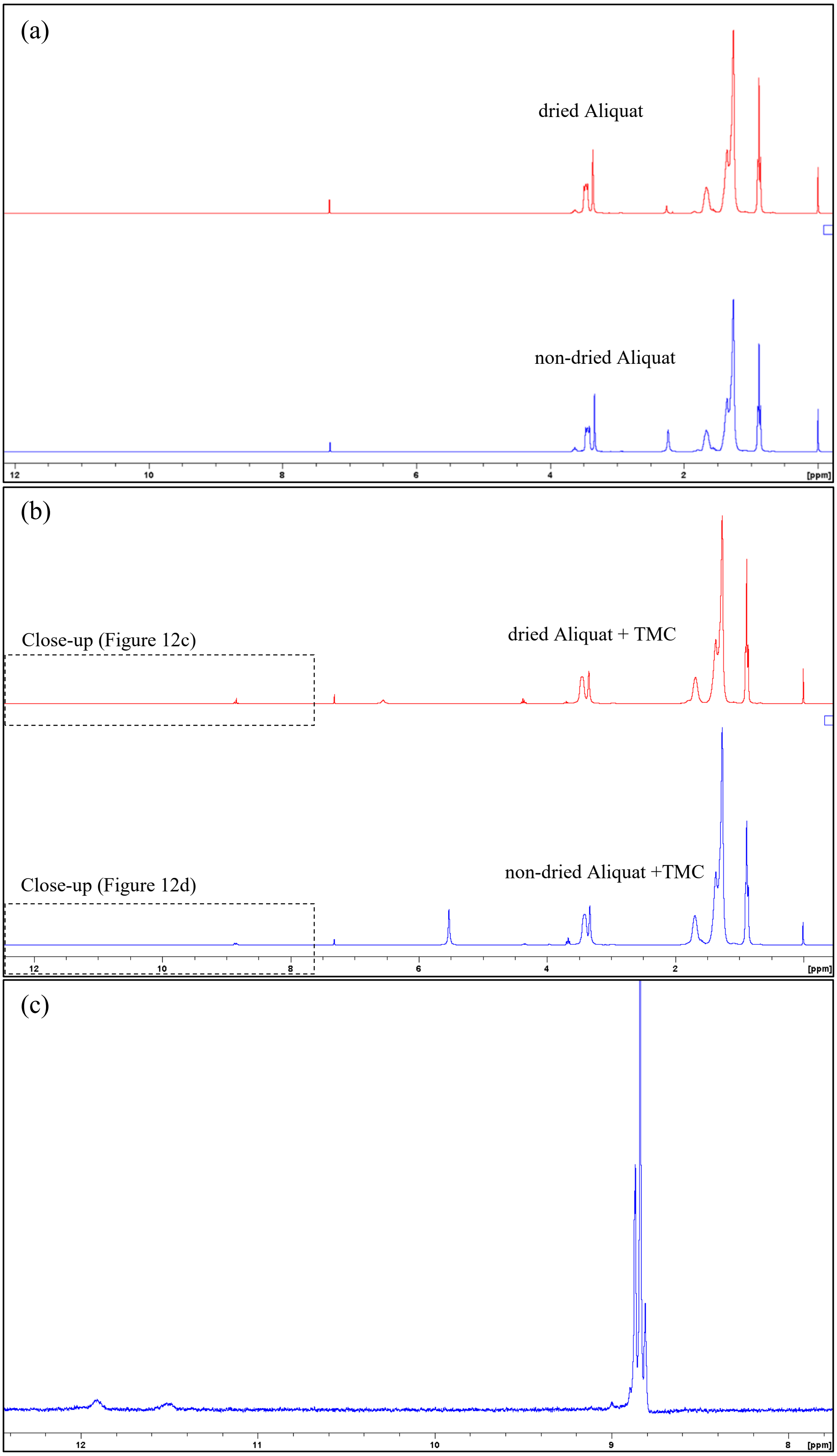
(a)
dried Aliquat
non-dried Aliquat
12
10
8
6
4
2
[ppm]
(b)
Close-up (Figure 12c)
dried Aliquat + TMC
Close-up (Figure 12d)
non-dried Aliquat +TMC
12
10
8
6
4
2
[ppm]
(c)
12
11
10
9
8
[ppm]

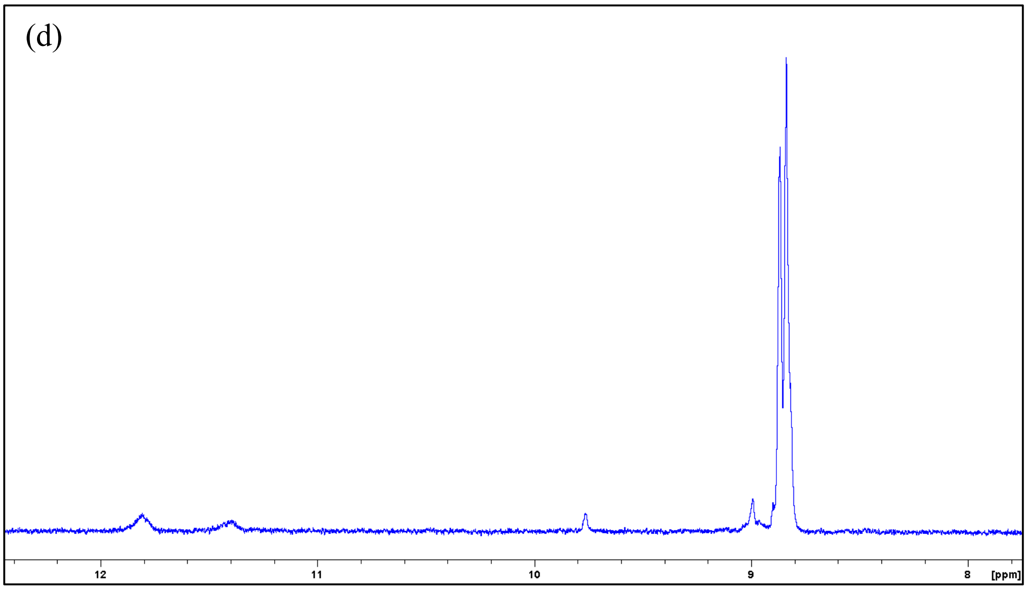

Figure S10. 300 MHz 1H-NMR spectra of (a) non-dried Aliquat (blue) and vacuum-dried at 80 °C (red), (b) 1.5 wt% TMC in dried (red) and non-dried (blue) Aliquat, (c) a close up of 1.5 wt% TMC in dried Aliquat and (d) a close up of 1.5 wt% TMC in non-dried Aliquat, in $CDCl_3$. On the x-axis, the chemical shift (ppm) is displayed.

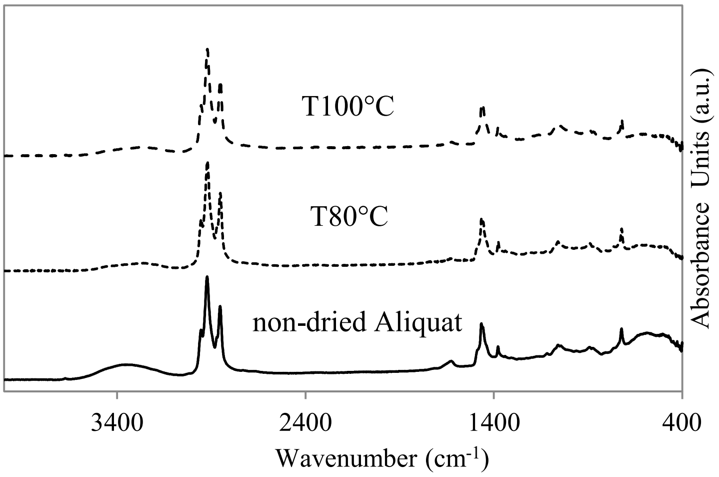

Figure S11. ATR-FTIR of Aliquat from bottle (full line), vacuum-dried at 80 °C (dotted line) and at 100 °C (striped line).

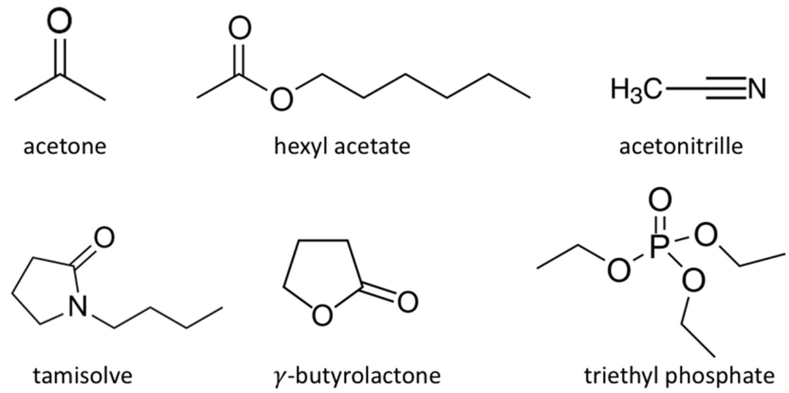

Figure S12. Structures of co-solvents.

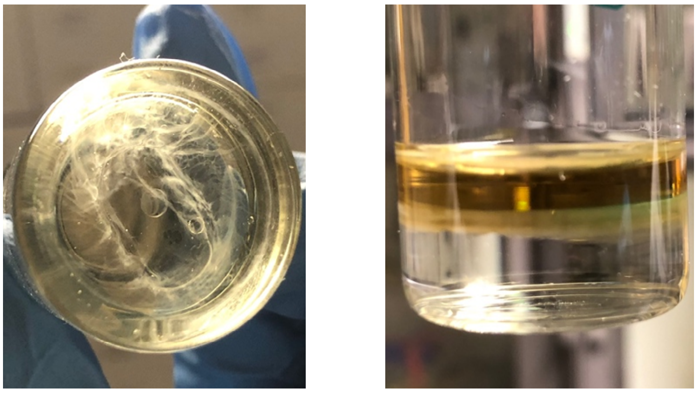

(a) (b)

Figure S13. Determination of cloudy structure when using 0.3 w/v% MPD in MilliQ water with 1.5 wt% TMC in (a) γ-butyrolacton, and (b) acetonitrile as co-solvents.

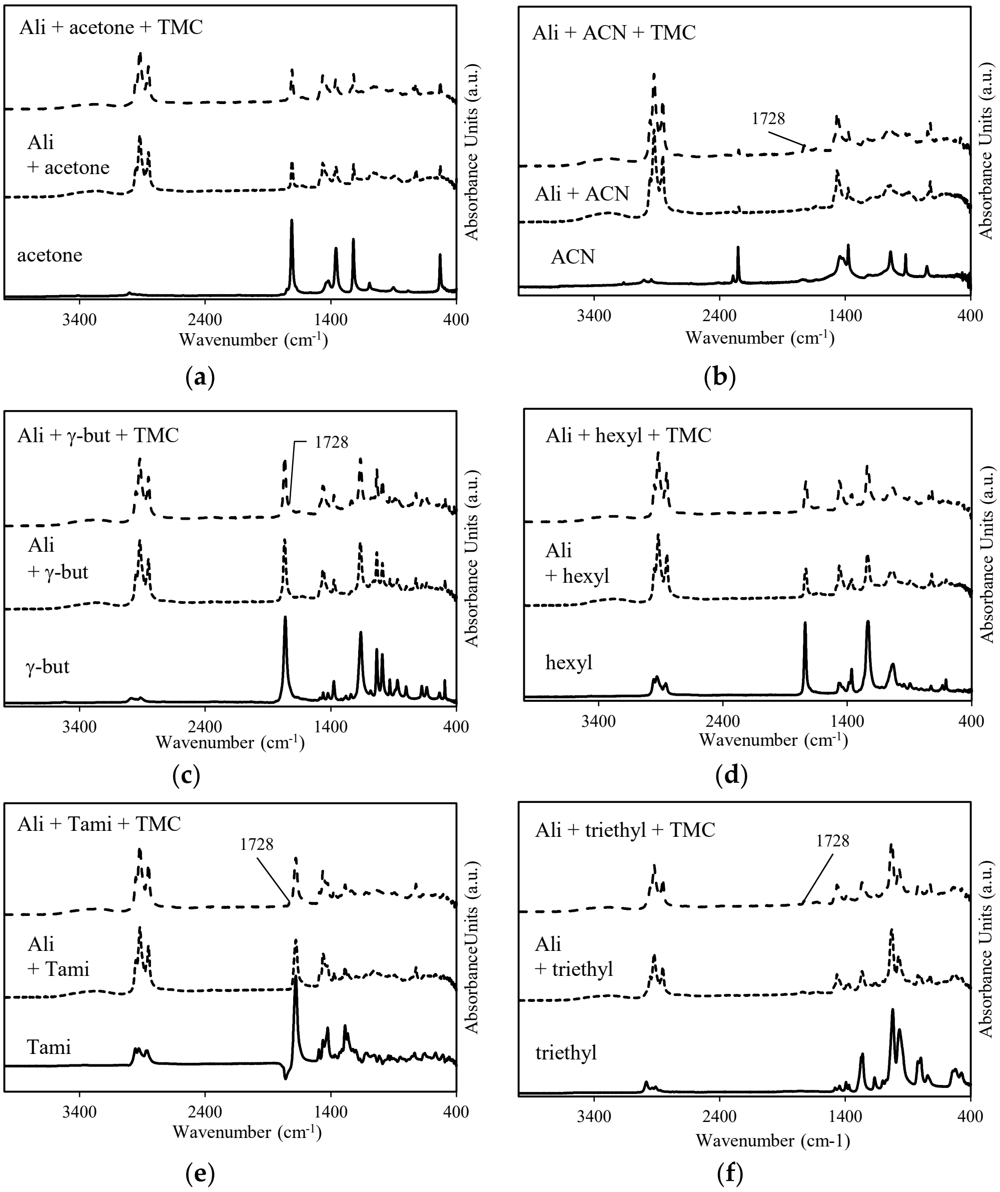

Figure S14. ATR-FTIR spectra of dried (100°C) Aliquat when 17.5 wt% co-solvents and/or 1.5 wt% TMC was added. ATR-FTIR spectra of pure co-solvent (full line), Aliquat (Al) + co-solvent (dotted

line) and Aliquat + co-solvent + TMC (striped line) for co-solvents (a) acetone, (b) acetonitrile (ACN), (c) γ-butyrolactone (γ-but), (d) hexyl acetate (hexyl), (e) Tamisolve® (Tami) and (f) triethyl phosphate (triethyl).

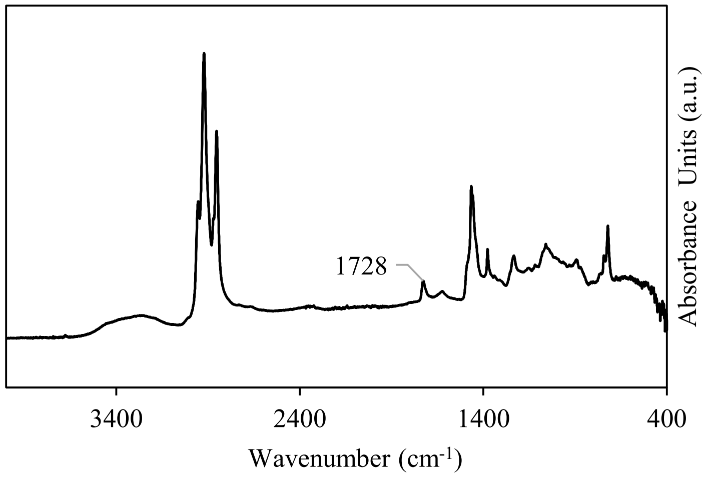

Figure S15. ATR FTIR spectrum of vacuum-dried Aliquat at 100 °C when 1.5 wt% TMC was added. An absorption band was observer at 1728 cm^{-1}, indicating hydrolysis and/or esterification of TMC.

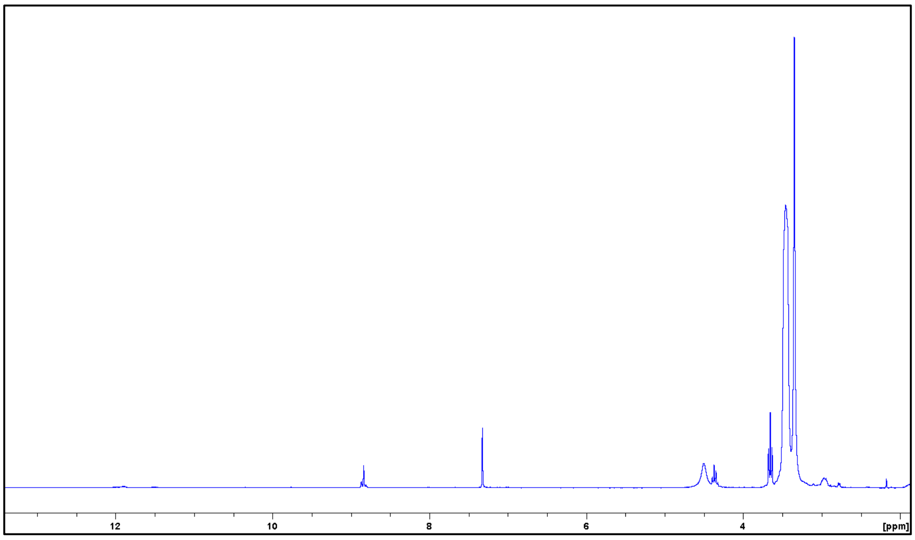

Figure S16. The 300 MHz ^{1}H-NMR spectra of vacuum dried Aliquat 336 at 100°C with 1.5 wt% TMC. On the x-axis, the chemical shift (in ppm) is displayed.

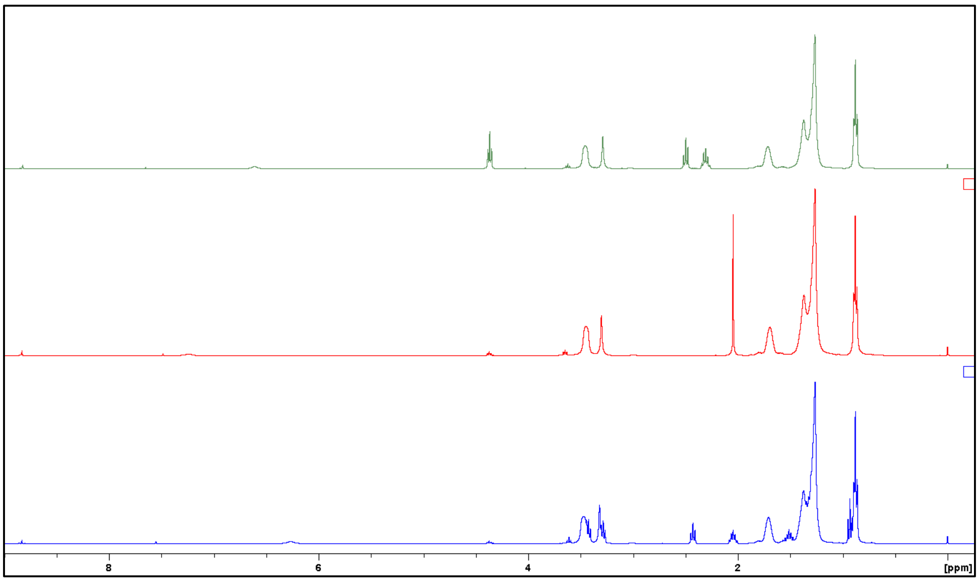

Figure S17. The 300 MHz ^{1}H-NMR spectra of 1.5 wt% TMC in vacuum dried Aliquat 336 at 100°C for 48h with 17.5 wt% γ-butyrolactone (green), acetonitrile (red) and Tamisolve® (blue).

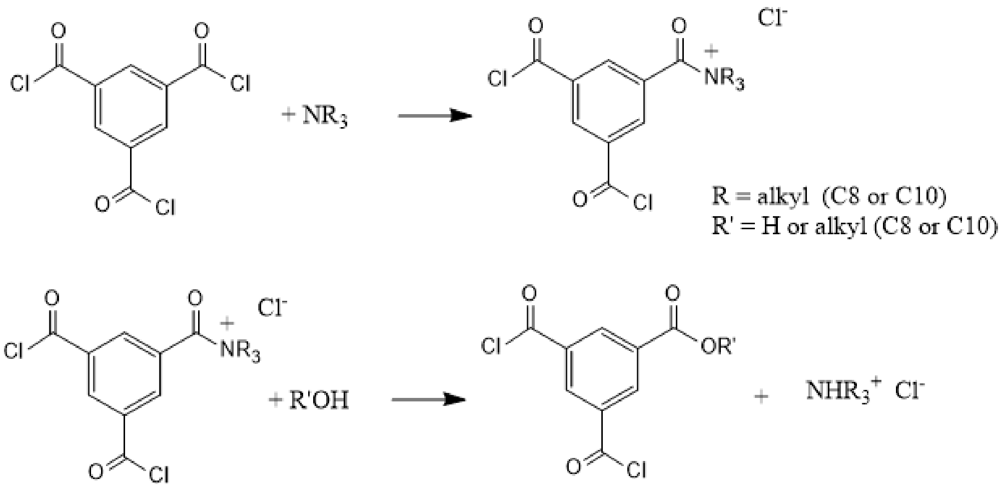

Figure S18. Possible reaction mechanisms of catalyzed hydrolysis (R′= H) and esterification (R′= alkyl) of TMC.

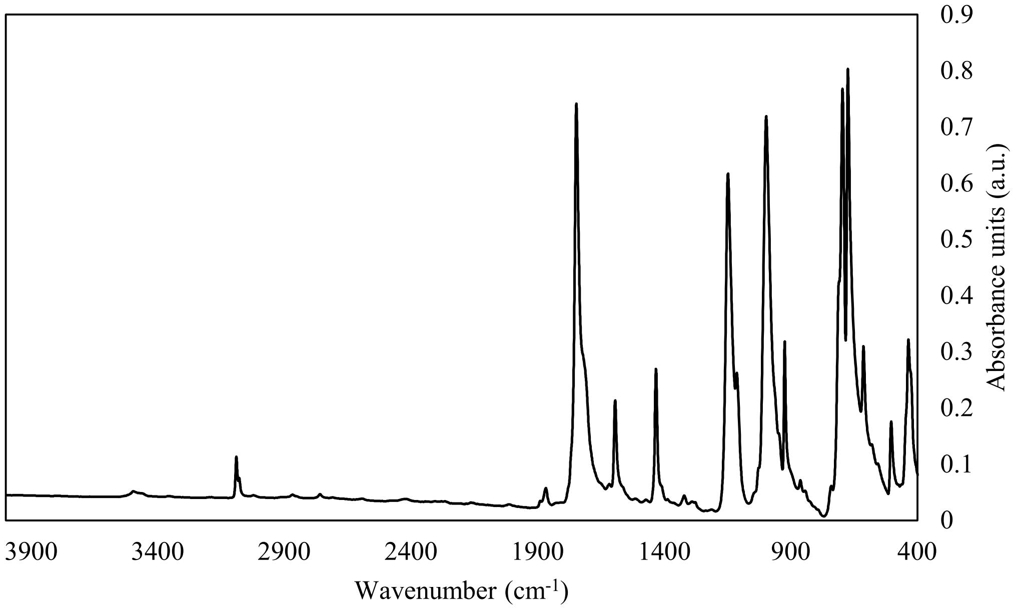

Figure S19. ATR-FTIR spectra of TMC in H_2O stirred for 1h.

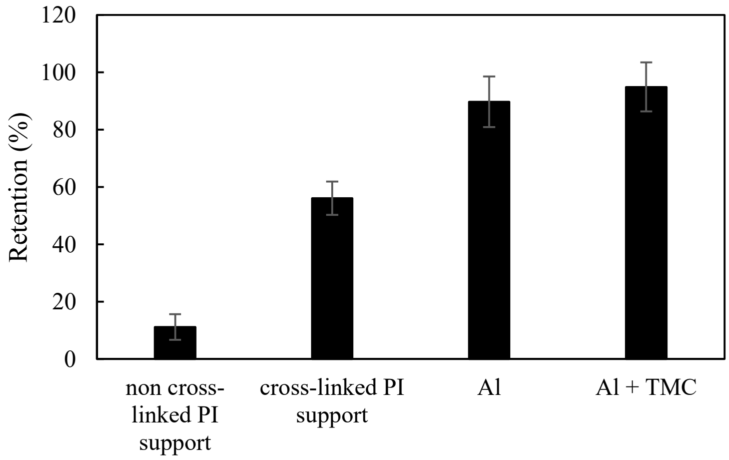

Figure S20. RB retentions of membranes with PI support, XL-PI support, Aliquat and Aliquat + 1.5 wt% TMC on XL-PI support.

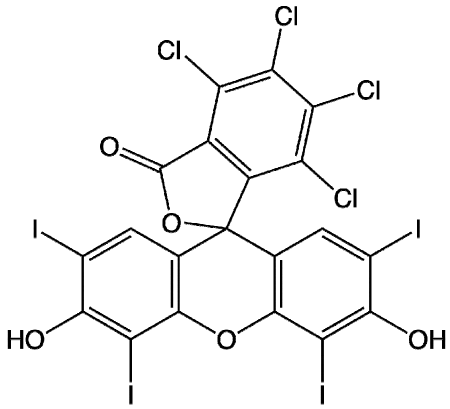

Figure S21. Structure of Rose Bengal.

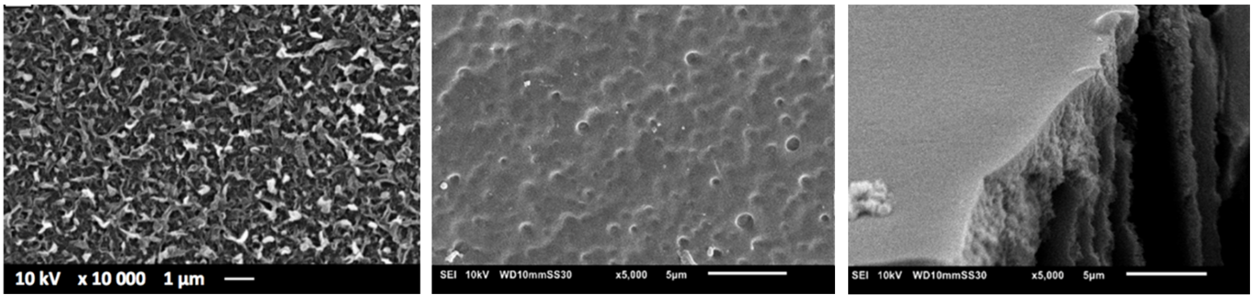

(**a**) (**b**) (**c**)

Figure S22. Surface SEM images of (a) typical ridge-and-valley morphology of an aromatic PA top-layer obtained with the conventional system (i.e. with hexane), (b) 1.51 wt% H_2O and (c) 21.3 wt% H_2O in Aliquat.

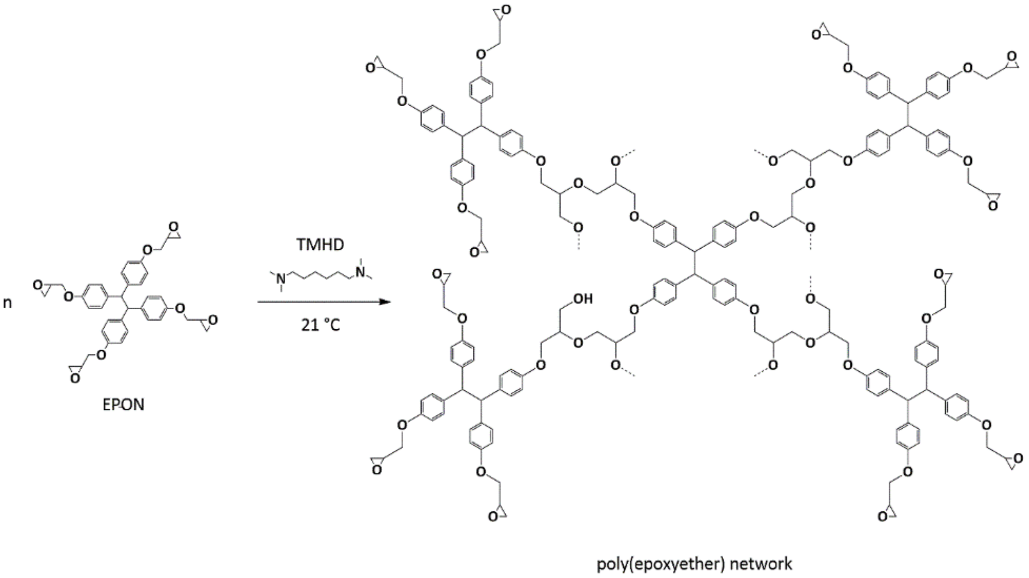

Figure S23. Anionic ring opening polymerization of EPON with TMHD (Adapted from [5]).

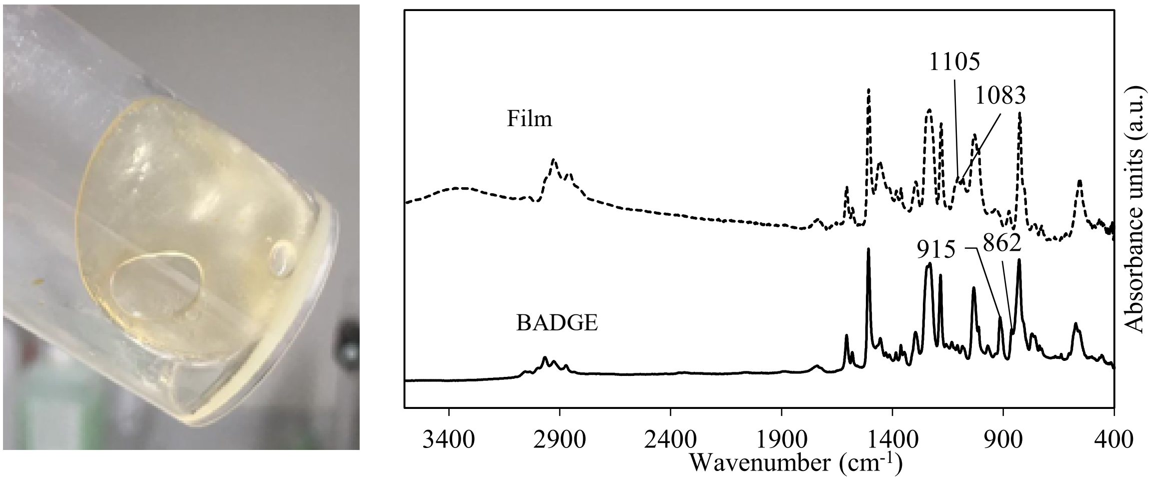

(a) **(b)**

Figure S24. (a) Successful isolation of a poly(β-alkanolamine) film. (b) ATR-FTIR spectra of isolated poly(β-alkanolamine) film (dotted line) and pure BADGE (full line).

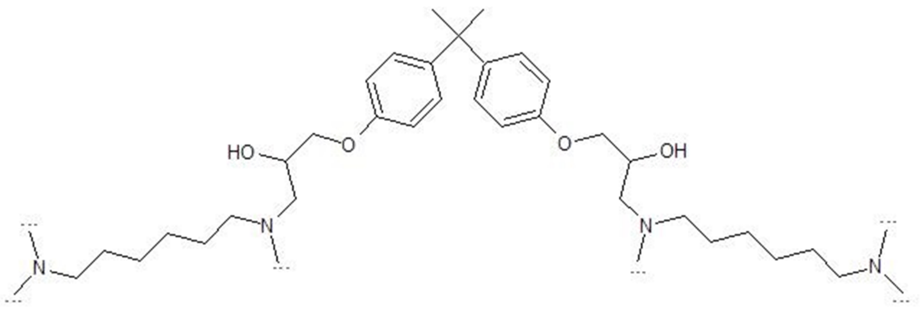

Figure S25. Structure of poly(β-alkanolamine).

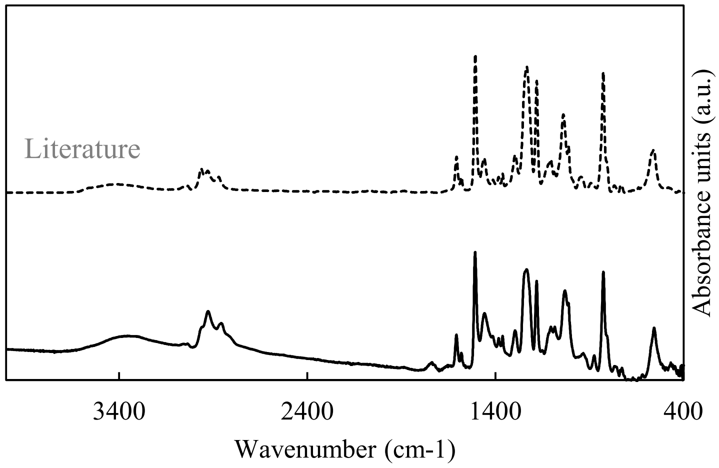

Figure S26. ATR-FTIR spectrum of the obtained poly(β-alkanolamine) film (full line) and literature data (dotted line) (adapted from [6]).

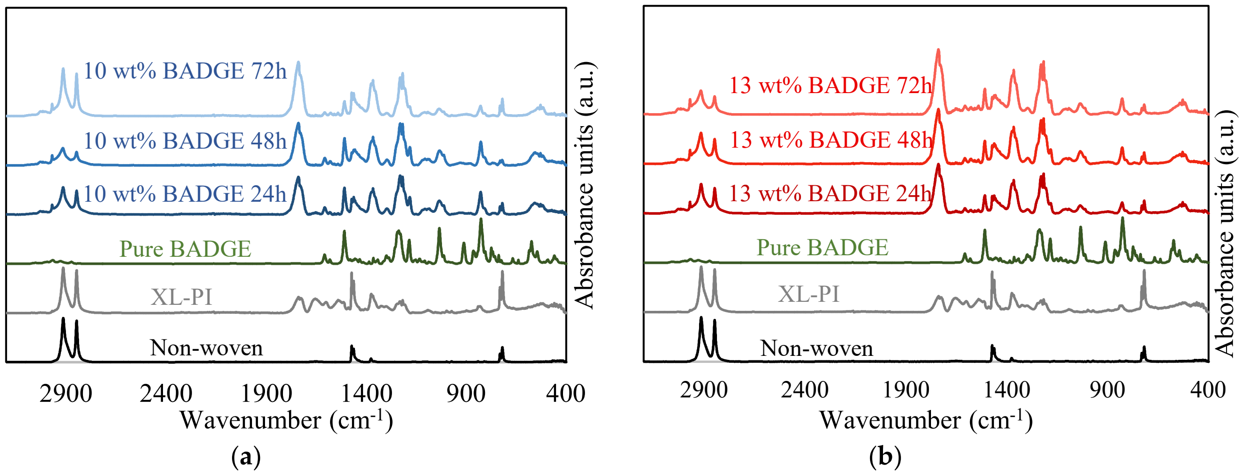

Figure S27. ATR-FTIR spectra for (a) 10 wt% HDA in water with 10 wt% BADGE in Aliquat and (b) 13 wt% HDA with 13 wt% E2.

Table S1. Prices of ILs.

	Price (€ per 100g)[a]
**[C_4mim][Tf_2N]**	226
**[C_4mpyr][Tf_2N]**	115
**Aliquat**	16

[a] For lab quantities

Table S2. Water content in dried co-solvents and Aliqaut/co-solvent mixtures.

Co-solvents and their mixtures with Aliquat	Water concentration before drying (ppm)	Water concentration after drying (ppm)
Hexyl acetate	294	/
Aliquat + hexyl acetate	/	1521
Tamisolve®	1365	/
Aliquat + Tamisolve®	/	419
Triethyl phosphate	1132	/
Aliquat + triethyl phosphate	/	NA
Acetonitrille	149	/
Aliquat + acetonitrille	/	332
γ-butyrolactone	182	/
Aliquat + γ-butyrolactone	/	188

Note: No measurement of triethyl phosphate/Aliquat due to practical infeasible (high viscosity).

Table S3. Preliminary screening of poly(β-alkanolamine) film formation for three different concentrations of HDA and BADGE.

Vial	Concentration	1 month at RT
1	2 wt% HDA + 0.1 wt% BADGE	-
2	10 wt% HDA + 0.5 wt% BADGE	-
3	10 wt% HDA + 10 wt% BADGE	-

Table S4. Vial tests for different concentrations of E2 and HDA.

Concentration	Reaction conditions (T & reaction time)	Images
7 wt% E2 & HDA	RT, 20 h	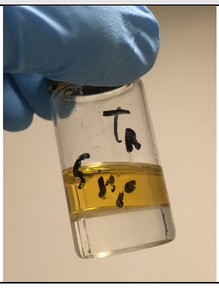

	RT, 80 h	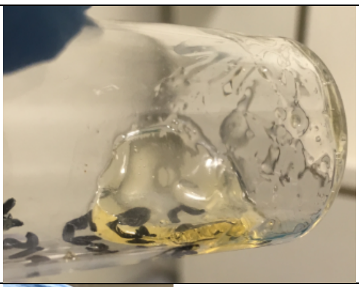
10 wt% E2 & HDA	RT, 20 h	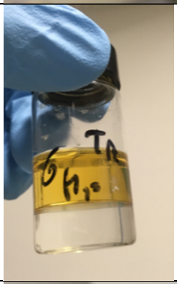
	80 °C, 20 h	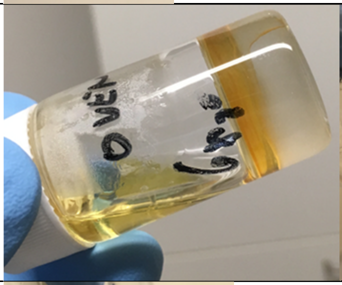 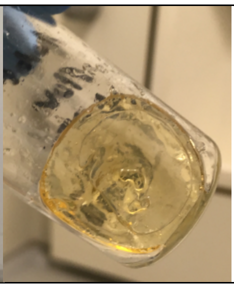
	RT, 80h	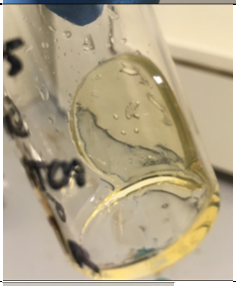
13 wt% E2 & HDA	RT, 20h	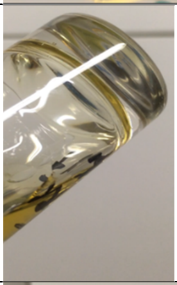
	80 °C, 20h	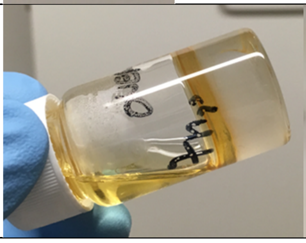 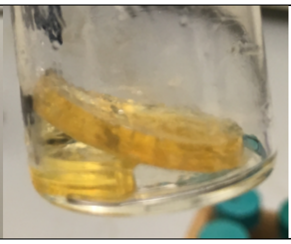

	RT, 80h	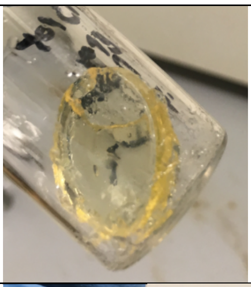
16 wt% E2 & HDA	RT, 20h	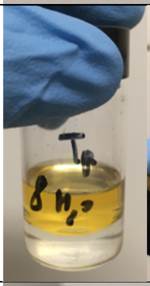 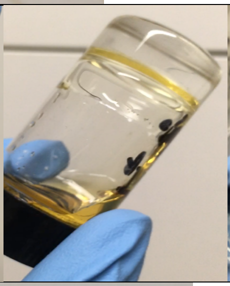 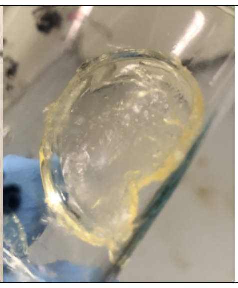
	80 °C, 20h	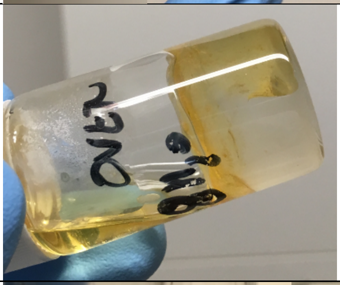
	RT, 80h	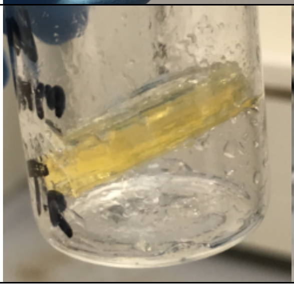 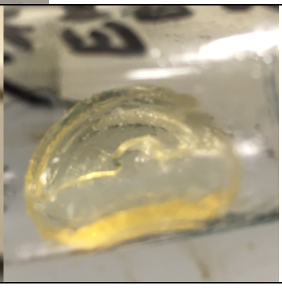

Table S5. Vial tests for different concentrations of E8 and HDA.

Concentration	Reaction condition (T & reaction time)	Images
2 wt% E8 & HDA	RT, 1 h	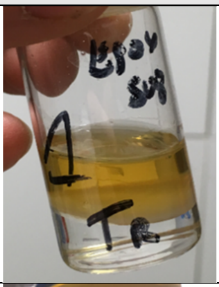
	RT, 20 h	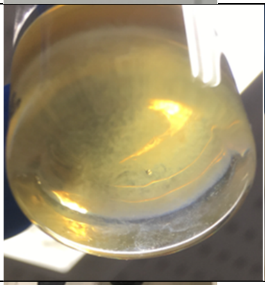 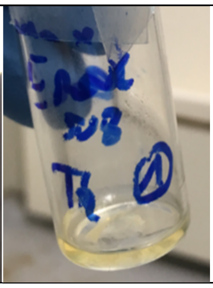
	80 °C, 20 h	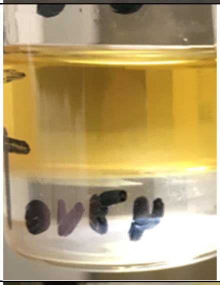
4 wt% E8 & HDA	RT, 1 h	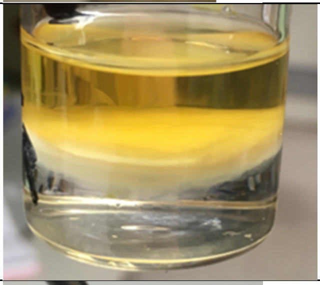
	80 °C, 1 h	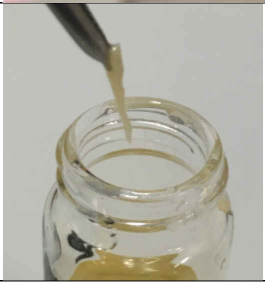

	RT, 20 h	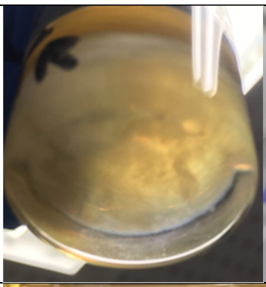 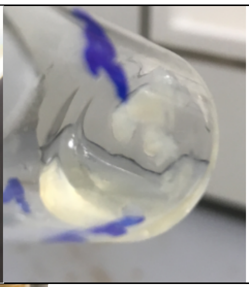
	80 °C, 20 h	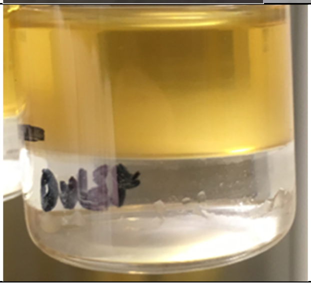

References

1. Hermans, S.; Mariën, H.; Dom, E.; Bernstein, R.; Vankelecom, I.F. Simplified synthesis route for interfacially polymerized polyamide membranes. *J. Membr. Sci.* **2014**, *451*, 148–156, doi:10.1016/j.memsci.2013.10.005.
2. Hermans, S.; Mariën, H.; Van Goethem, C.; Vankelecom, I.F. Recent developments in thin film (nano)composite membranes for solvent resistant nanofiltration. *Curr. Opin. Chem. Eng.* **2015**, *8*, 45–54, doi:10.1016/j.coche.2015.01.009.
3. Vanherck, K.; Cano-Odena, A.; Koeckelberghs, G.; Dedroog, T.; Vankelecom, I. A simplified diamine crosslinking method for PI nanofiltration membranes. *J. Membr. Sci.* **2010**, *353*, 135–143, doi:10.1016/j.memsci.2010.02.046.
4. Mariën, H.; Vankelecom, I.F. Optimization of the ionic liquid-based interfacial polymerization system for the preparation of high-performance, low-fouling RO membranes. *J. Membr. Sci.* **2018**, *556*, 342–351, doi:10.1016/j.memsci.2018.03.071.
5. Verbeke, R.; Arts, W.; Dom, E.; Dickmann, M.; Egger, W.; Koeckelberghs, G.; Szymczyk, A.; Vankelecom, I.F. Transferring bulk chemistry to interfacial synthesis of TFC-membranes to create chemically robust poly(epoxyether) films. *J. Membr. Sci.* **2019**, *582*, 442–453, doi:10.1016/j.memsci.2019.02.016.
6. Cabanelas, J.C.; Baselga, J. Applications of FTIR on Epoxy Resins - Identification, Monitoring the Curing Process, Phase Separation and Water Uptake. In *Infrared Spectroscopy - Materials Science, Engineering and Technology*; InTech, 2012.
